# Organic AIE Nanoradiosensitizer Potentiates X‐Ray Triggered Continuous Reactive Oxygen Species Generation for Potent Cancer Radioimmunotherapy

**DOI:** 10.1002/adma.202502898

**Published:** 2025-06-19

**Authors:** Qingyong Xu, Minmin Zhang, Qinqin Huang, Song Gao, Sitong Chu, Qi Li, Wanyao Chen, Xinglong Zhang, Tianfu Zhang, Ben Zhong Tang, Shipeng Ning

**Affiliations:** ^1^ Department of Breast Radiotherapy Harbin Medical University Cancer Hospital Harbin Heilongjiang 150081 P. R. China; ^2^ Department of Breast Surgery Harbin Medical University Cancer Hospital Harbin Heilongjiang 150081 P. R. China; ^3^ Department of Breast and Thyroid Surgery Liuzhou People's Hospital Liuzhou Guangxi 545006 P. R. China; ^4^ The Research and Application Center of Precision Medicine The Second Affiliated Hospital Zhengzhou University Zhengzhou Henan 450014 P. R. China; ^5^ Department of Breast Surgery The Second Affiliated Hospital of Guangxi Medical University Nanning Guangxi 530000 P. R. China; ^6^ Research Center of Nanomedicine Technology The Second Affiliated Hospital of Guangxi Medical University Nanning Guangxi 530000 P. R. China; ^7^ Guangzhou Institute of Cancer Research the Affiliated Cancer Hospital School of Biomedical Engineering Guangzhou Medical University Shenzhen Guangdong 511436 P. R. China; ^8^ School of Science and Engineering Shenzhen Institute of Aggregate Science and Technology The Chinese University of Hong Kong (CUHK‐Shenzhen) Shenzhen Guangdong 518172 P. R. China

**Keywords:** aggregation‐induced emission, continuous ROS generation, macrophage polarization, organic radiosensitizer, radioimmunotherapy

## Abstract

Organic radiosensitizers (ORSs), with structural versatility, hold promise for sensitizing radiotherapy (RT) by interacting with X‐ray photons to generate reactive oxygen species (ROS). They often consist of low‐Z elements that have inherently low X‐ray deposition capability. However, current ORSs suffer from short circulation half‐lives and low tumor retention, contradicting their requirements for sustained ROS generation upon RT in tumor sites. Herein, a glutathione‐responsive system is prepared, loaded with aggregation‐induced emission molecules (TPEPy‐I) and Ferriprotoporphyrin IX chloride (Hemin), to act as an ORS (named THN) for X‐ray triggered sustained ROS generation for efficient antitumor immunotherapy. In detail, THN effectively deposited X‐ray photons, which interact with water molecules to generate abundant ROS under external radiation. Subsequently, THN‐mediated radiosensitization stimulated tumor cells to produce hydrogen peroxide (H_2_O_2_) by upregulating NOX4 protein, promoting the chemodynamic process of Hemin reacting with H_2_O_2_ to continuously produce hydroxyl radicals. The double‐promoting ROS generation of THN induced massive immunogenic cancer cell death and polarized M2 macrophages into the M1 phenotype for enhanced antitumor immunotherapy. Experiments revealed that THN‐sensitized RT inhibited tumor recurrence and increased memory T‐cell proportion for long‐term antitumor immunity. The developed ORS has great clinical potential as both a radiosensitizer and a post‐RT immunomodulatory agent.

## Graphical abstract

Herein, a glutathione‐responsive system (named THN) is prepared for X‐ray‐triggered sustained ROS generation for efficient antitumor immunotherapy. The double‐promoting ROS generation of THN induced massive immunogenic cancer cell death and polarized M2 macrophages into the M1 phenotype for enhanced antitumor immunotherapy. Experiments revealed that THN‐sensitized RT inhibited post‐surgery tumor recurrence and increased memory T‐cell proportion for long‐term antitumor immunity.

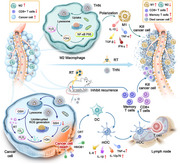

## 1 Introduction

In current clinical cancer therapy, half of all patients are treated with radiotherapy (RT) either alone or combined with other types of treatments.^[^ ^1^ ^]^ X‐rays possess deeper tissue penetration than light energy, facilitating the delivery of high‐energy photons into the human body and producing abundant reactive oxygen species (ROS). However, the radioresistance of tumor cells remains a challenging issue, leading to treatment failure and even cancer recurrence. Recently, there has been a great interest in developing nanomaterials‐based radiosensitizers to enhance radiosensitivity and overcome radioresistance.^[^ ^1^, ^2^ ^]^ Most radiosensitizers developed so far are based on inorganic or high atomic numbers (e.g. high‐Z metals and rare earth metals) nanomaterials, which may cause undesirable adverse effects.^[^ ^3^ ^]^ Organic radiosensitizers (ORSs) exhibited structural multifunctionality and showed promise in interacting with X‐ray via several physical processes, releasing auger electrons, Compton electrons, and photoelectrons, which interact with water molecules to generate ROS.^[^ ^1^, ^3^, ^4^ ^]^ Nonetheless, organic compounds are mainly composed of low‐atomic‐number elements, which have low absorption rates for X‐rays and are difficult to induce RT effects. Therefore, efficient ORSs should be rationally designed to sensitize RT for cancer treatment.

Recent studies have established that iodinated contrast agents can enhance X‐ray radiation‐induced cancer cell death. For example, potassium iodide nanoparticles have been studied to augment RT.^[^ ^5^ ^]^ However, iodinated contrast agents exhibit short circulation half‐lives and low tumor retention.^[^ ^6^ ^]^ Inspired by those clinical findings, introducing iodine atoms into certain organic luminescent materials can potentially improve radiosensitivity. Yet, the efficacy remains limited by the low accumulation of iodine inside cancer cells. Thus, a universal method is urgently needed to increase the conversion rate of X‐ray photons to ROS and ensure its continuous production.^[^ ^1a^ ^]^ While X‐ray‐induced afterglow and radiodynamic therapy by using radio afterglow agents tackle the tissue penetration issue and facilitate long‐term ROS generation, they are limited to a few choices of rare earth metal‐based inorganic nanoparticles that display uncontrollable “always on” radiodynamic theranostic functions.^[^ ^7^ ^]^ How to precisely control the sustained production of ROS induced by X‐ray irradiation is a key issue in the ORSs system. Recent studies have reported that RT disrupts the inherent redox homeostasis of tumor tissues and stimulates tumor cells to produce hydrogen peroxide (H_2_O_2_) by upregulating NOX4 protein.^[^ ^8^ ^]^ Building upon the discovery, we hypothesize that the combination of ORSs with H_2_O_2_‐mediated chemodynamic therapy could potentially enhance a long‐lasting radiosensitizing effect for efficient RT‐radiodynamic therapy.

**Scheme 1:**
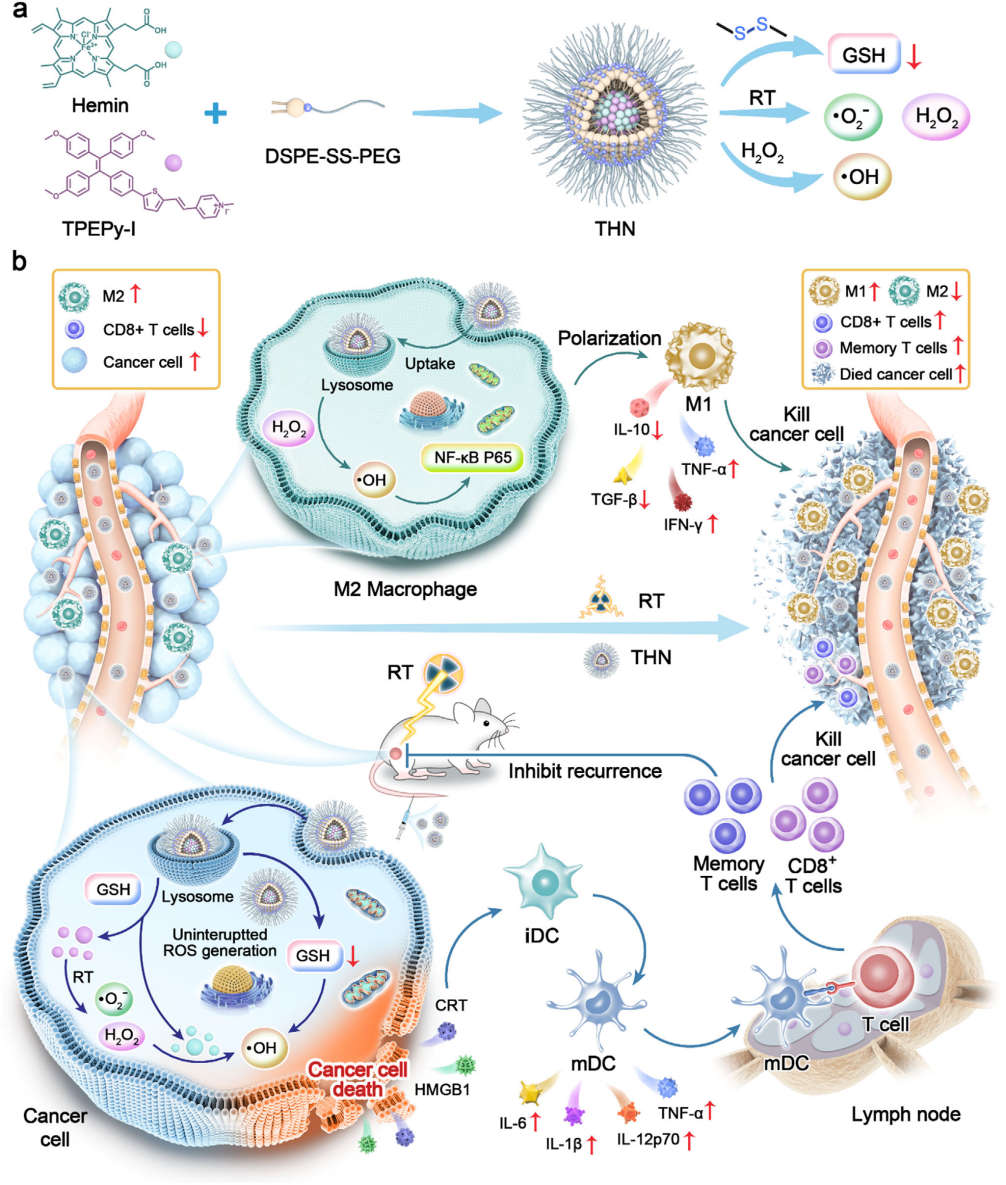
Schematic illustration of organic nano‐radiosensitizer based on AIEgens for X‐ray triggered continuous ROS generation in tumors and efficient radioimmunotherapy. a) Preparation and function of THN. b) The anti‐tumor mechanism of THN.

In this study, a glutathione (GSH)‐responsive ORSs system (named THN) was prepared for X‐ray‐induced sustained ROS generation and efficient antitumor radioimmunotherapy (**Scheme** **1**). THN was prepared by encapsulating aggregation‐induced emission (AIE) molecules (named TPEPy‐I; full name: 1‐methyl‐4‐[2‐[5‐[4‐[1,2,2‐tris(4‐methoxyphenyl) ethenyl] phenyl]‐2‐thienyl] ethenyl]‐Pyridinium iodide) and Ferriprotoporphyrin IX chloride (Hemin) with 1,2‐Distearoyl‐sn‐glycero‐3‐phosphoethanolamine‐disulfide bond‐Polyethylene Glycol (DSPE‐SS‐PEG) through the solvent emulsification volatilization method. Upon accumulation at the tumor site via systemic circulation and rapidly releasing TPEPy‐I and Hemin, THN exhibited high specificity to the GSH‐overexpress tumor microenvironment. Under external X‐ray irradiation, TPEPy‐I effectively deposited X‐ray photons and promoted enormous ROS conversion. Simultaneously, its radiosensitizing effect stimulates tumor cells to produce H_2_O_2_, which is beneficial for enhancing the chemodynamic processes of Hemin to catalyze H_2_O_2_ and continuously generate hydroxyl radicals (•OH). The results of in vitro and in vivo experiments indicated that the THN system‐induced double‐jump ROS production triggered extensive immunogenic cell death (ICD) in tumor cells and polarized M2 macrophages into M1 phenotype, relieving the immunosuppressive microenvironment after RT and activating the antitumor immune ability of CD8^+^ T cells. Moreover, THN‐sensitized RT can promote the proportion of memory T cells, creating long‐term antitumor immunity to inhibit post‐surgical tumor recurrence. Therefore, THN not only serves as an ORS but also a tumor immunomodulatory agent to enhance radioimmunotherapy and holds immense potential for clinical translation.

## 2 Results and Discussion

### 2.1 Preparation and Characterization of THN

**Figure 1:**
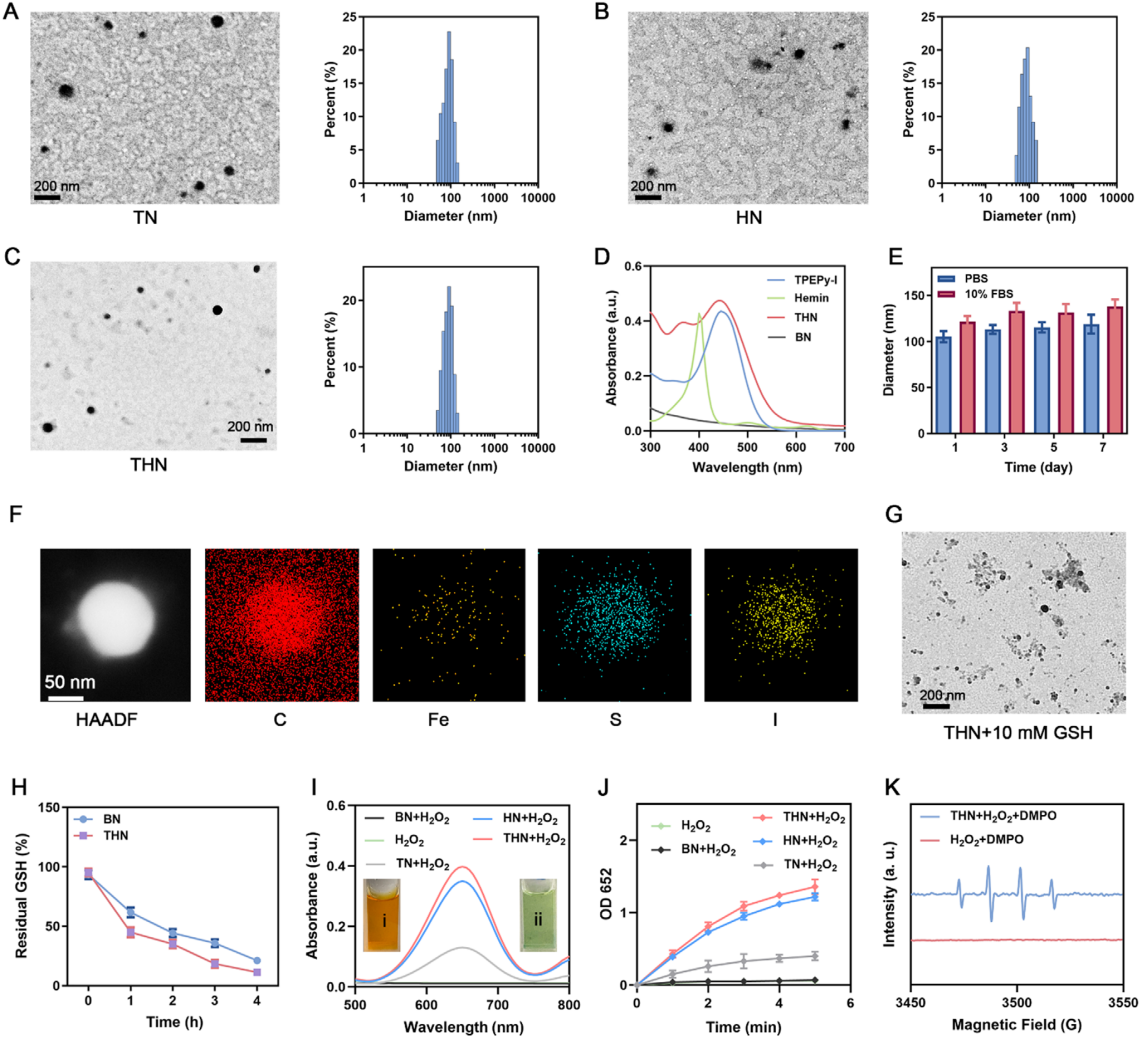
TEM image and diameter distribution of (A) TN, B) HN, and C) THN. D) Absorption spectra of TPEPy‐I in DMSO, Hemin in DMSO, THN in PBS, and BN (bare nanoparticles without drug loading in PBS). E) Stability of THN in various solutions at different times. F) HAADF‐SETM images and element mapping of THN. G) TEM image of THN after 10 mm GSH treatment for 4h. H) GSH residual rates at different times of incubation with BN or THN. I) Oxidation and J) Absorbance change at 652nm of TMB due to generated •OH by indicated treatments. i: THN in PBS, ii: THN+TMB+H_2_O_2_. K) ESR spectra showing the •OH of THN with H_2_O_2_ (25 µm).

TPEPy‐I was initially prepared^[^ ^6a^ ^]^ (Scheme S1, Supporting Information), and its characterization using nuclear magnetic resonance (NMR) and high‐resolution mass spectrometry (HRMS) is presented in Figures S1–S3 (Supporting Information). The molecule exhibited obvious AIE characteristics, and its luminescence was substantially enhanced in the aggregated state (Figure S4, Supporting Information). Then, TPEPy‐I and Hemin were loaded into GSH‐responsive DSPE‐SS‐PEG2000 nanoparticles, termed THN, using a nanoprecipitation method.^[^ ^9^ ^]^ To set up control groups, samples loaded only with TPEPy‐I or Hemin were prepared using the same method: TPEPy‐I@DSPE‐SS‐PEG nanoparticles (TN) and Hemin@DSPE‐SS‐PEG nanoparticles (HN). The transmission electron microscopy (TEM) images and particle size distributions of TN, HN, and THN are depicted in **Figure** **1**A–C, revealing their uniform spherical morphologies with diameters of ≈100 nm. Additionally, the polydispersity index (PDI) values measured for THN (0.155), TN (0.164), and HN (0.162) were all below 0.2, indicating uniform distribution of the nanoparticles. UV–vis absorption spectra were obtained for TPEPy‐I in dimethyl sulfoxide (DMSO), Hemin in DMSO, BN (bare nanoparticles without drug loading) in phosphate‐buffered saline (PBS), and THN in PBS (Figure 1D). In the DMSO solution, TPEPy‐I and Hemin showed maximum absorption peaks at 445 and 400 nm, respectively. The absorption peak of THN demonstrated a slight blue shift, indicating the occurrence of H‐aggregation between molecules.^[^ ^10^ ^]^ The encapsulation efficiency of THN for TPEPy‐I and Hemin was 26.64% and 10.53%, respectively. THN exhibited good stability in PBS and 10% fetal bovine serum solutions (Figure 1E). In addition, high‐angle annular dark‐field scanning transmission electron microscopy (HAADF‐STEM) and elemental maps of THN confirmed the even distribution of C, S, I, and Fe elements within the nanoparticles (Figure 1F). Disulfide bonds are believed to be used to deplete GSH and induce ferroptosis in tumor cells.^[^ ^11^ ^]^ In the presence of disulfide bonds, the GSH responsiveness of THN was evaluated to assess the GSH‐triggered controlled drug release. After incubating with 10 mm GSH, TEM of THN shows irregular particle sizes and partial loss of spherical morphology, revealing the disassembly of THN (Figure 1G). In addition, Both BN and THN exhibit good GSH‐depleting capacity (Figure 1H). Under X‐ray irradiation, little change was noted in THN morphology (Figure S5, Supporting Information), indicating its stability under RT. As shown in Figure S6 (Supporting Information), we co‐incubated the lysates of different cells with THN and found that the lysates of tumor cells could cause THN disintegration, while the lysates of macrophages did not cause THN disintegration. It indicates that THN can be selectively activated. To testify to the chemodynamic effect of THN, Tetramethyl‐benzidine (TMB) was used to measure •OH generation during the Fenton reaction.^[^ ^12^ ^]^ After oxidation by •OH, the color of the solution mixture changed from colorless to bluish‐green, with a maximum absorption at 652 nm. These results demonstrated that HN and THN‐loaded Hemin, can catalyze H_2_O_2_ and generate •OH (Figure 1I,J). The interaction between TN and H_2_O_2_ also induced TMB coloration, possibly because of the redox reaction between iodide ions and H_2_O_2_. Furthermore, using 5, 5‐dimethyl‐1‐pyrroline N‐oxide (DMPO) as a spin‐trapping agent, the catalytic activity of THN was proven using electron spin resonance (ESR) spectra (Figure 1K).

### 2.2 Theoretical Simulation Method and ROS Generation under X‐Ray Irradiation

In this study, the Geant4 Monte Carlo toolkit (version G4.10.07) was used for microdosimetric particle transport simulations.^[^ ^13^ ^]^ The Geant4‐DNA extension was utilized to calculate energy deposition in water, while the Penelope Low Energy Package was applied to model interactions within nanoparticles.^[^ ^14^ ^]^ To account for multiple scatterings of electrons and ions, the G4 Urban model was implemented with a 1 nm step cut. Additionally, atomic de‐excitation processes, including fluorescence and Auger electron emission, were enabled in all simulations.

**Figure 2:**
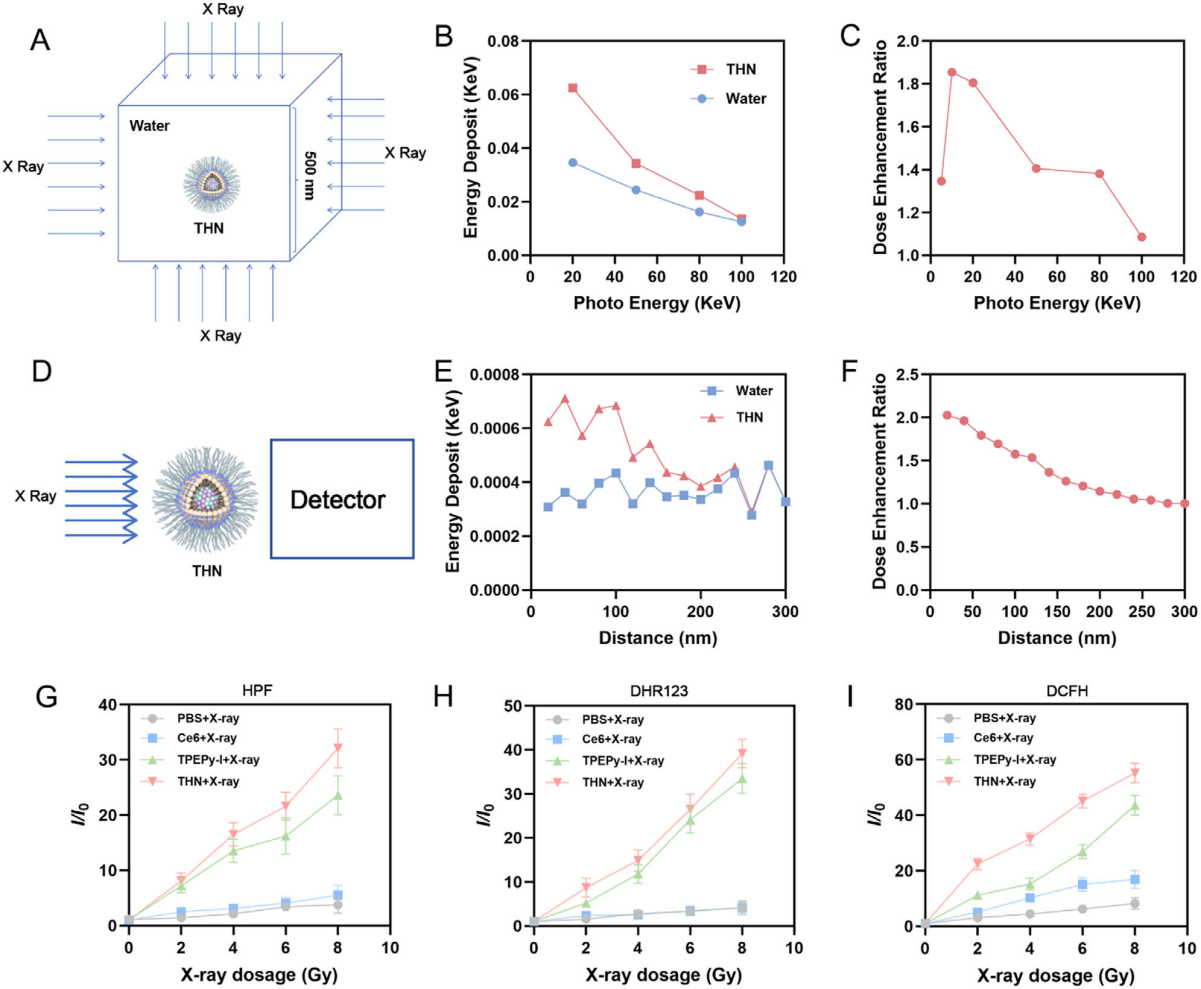
A) Schematic illustration of energy‐dependent simulations. B) Energy deposit of THN or water under different photo energy X‐ray irradiation and C) corresponding dose enhancement ratio (DER). D) Schematic illustration of distance‐dependent simulations. E) Energy deposition and F) DER at different distances from THN or water under X‐ray irradiation. Measurement of G) hydroxyl radical, H) superoxide anion, and I) total ROS generation with fluorescence enhancement of corresponding indicator for Ce6 (in DMSO/water mixture, *fw* = 99%), TPEPy‐I (in DMSO/water mixture, *fw* = 99%) and THN upon X‐ray irradiation with different dosage. The concentration of TPEPy‐I and Ce6 is 50 µg mL^−1^.

For energy‐dependent simulations, the physical radiosensitization efficiency of a single nanoparticle under different monoenergetic X‐ray irradiations was investigated (**Figure** **2**A). In the control group without THN (pure water), THN can deposit more energy under X‐ray irradiation (Figure 2B,C), confirming its radiosensitizing ability. It is worth noting that as the energy of the incident electrons increases, the difference in the deposition of THN and water decreases. This is because high‐energy X‐rays are more likely to penetrate the cube set by the model without being captured by THN. For distance‐dependent simulations, the nanoparticles were placed at the center of a water medium, and the dose enhancement ratio (DER) was calculated under small‐animal irradiation conditions (The X‐ray was generated by a tungsten target with a peak energy of 100 keV, which was also simulated using Geant4) at varying distances (Figure 2D). As the distance increases, the deposition energy around THN decreases, while in short distances, the deposition energy of THN is higher than that of water (Figure 2E,F). This result further validated its radiosensitizing ability. A critical indicator of radiosensitizers is the capacity to generate ROS during radiotherapy.^[^ ^1^, ^15^ ^]^ Subsequently, we validated the differential ROS generation capacity of THN under radiotherapy using fluorescent probes. As shown in Figure 2G–I and Figure S7 (Supporting Information), THN and TPEPy‐I produce more ROS, including •OH and •O_2_^−^ under X‐ray irradiation compared to Ce6, indicating their excellent radiosensitizing effects. Furthermore, TPEPy‐I+Hemin in the dispersed state or GSH pretreatment has minimal impact on the total ROS production in the THN+RT (Figure S8, Supporting Information).

### 2.3 Radiosensitizing Effect of THN for In Vitro Anticancer

**Figure 3:**
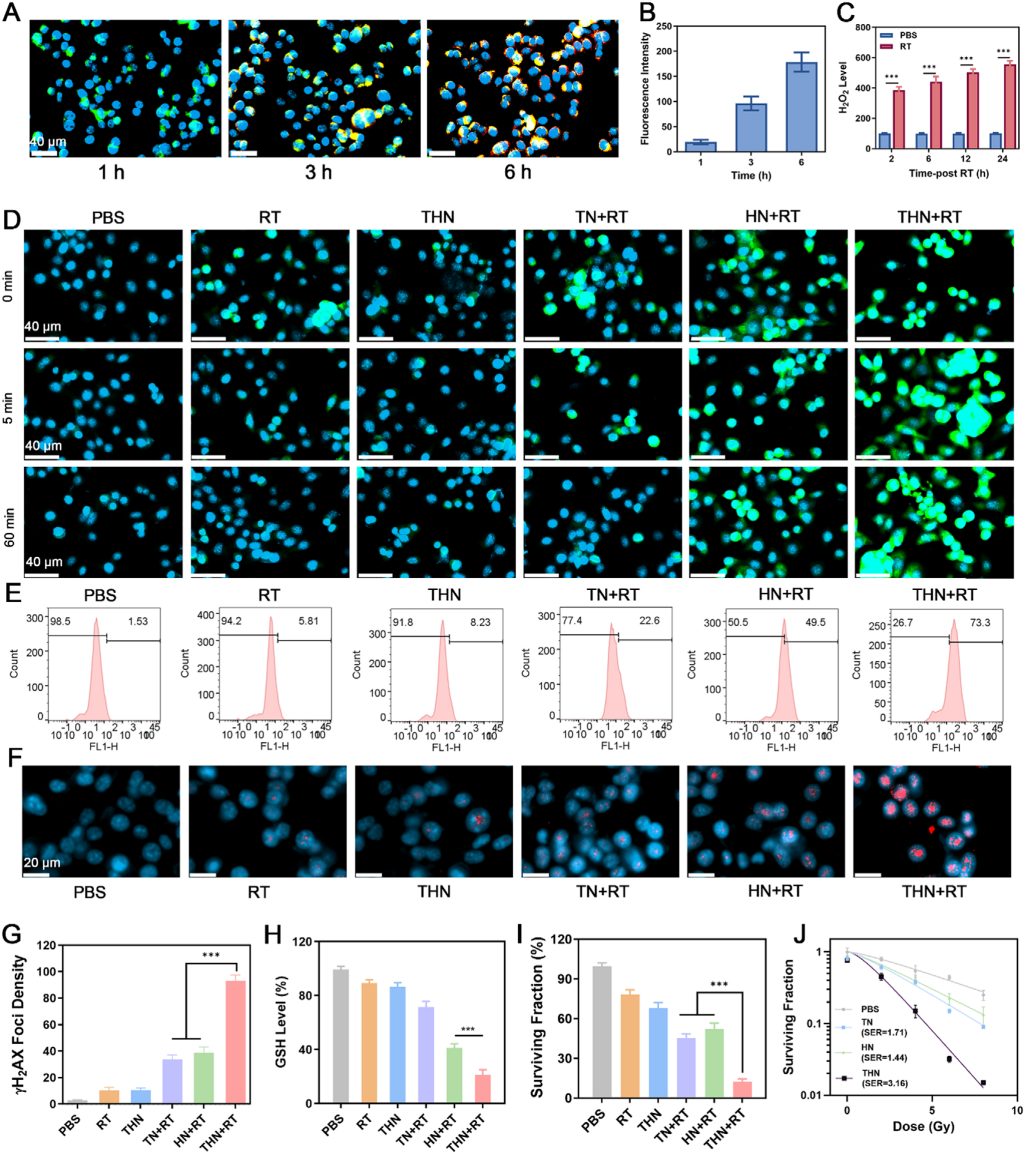
A) Confocal laser scanning microscopy (CLSM) images and B) quantitative red fluorescence intensity of 4T1 cancer cells treated with labeled THN. DAPI: blue; TPEPy‐I: red; Lyso‐Tracker Green: green. THN concentration: 10 µg mL^−1^. C) Production of intracellular H_2_O_2_ by RT. D) CLSM images and of •OH (green fluorescence) generated in 4T1 cells at different times after treatments. E) •OH fluorescence intensity of Figure 2D (60 min). F) Expression of γ‐H_2_AX (red fluorescence) and G) γ‐H_2_AX Foci density from 4T1 cells following treatment with different formulations. RT: 4Gy. H) The intracellular GSH content after 60 min of different treatments. I) Clonogenic survival assay of 4T1 cells treated with different formulations. J) Clonogenic survival assay of 4T1 cells treated with different formulations under a series of radiation doses at 0, 2, 4, 6, and 8 Gy. TPEPy‐I concentration: 100 µg mL^−1^. Data are represented as mean ± standard deviation (SD). Statistical significance was calculated via one‐way ANOVA with Tukey's test: ^***^ *p* < 0.001.

The cellular uptake of THN was examined using confocal laser scanning microscopy (CLSM). Considerable TPEPy‐I fluorescence was noted in 4T1 cells with increasing incubation time of THN, indicating that they can be effectively engulfed by tumor cells (**Figure** **3**A,B). We then detected the production of ROS in macrophages after THN treatment, as shown in Figure S9A (Supporting Information). After treatment with a higher concentration of THN, obvious ROS generation was observed in macrophages. THN exhibited low toxicity toward macrophages, suggesting good biocompatibility (Figure S9B, Supporting Information). RT was then observed to upregulate NADPH oxidase 4 (NOX4) expression and elicit H_2_O_2_ generation (Figure 3C; Figure S10, Supporting Information). Previous studies have documented that RT‐induced tumor hypoxia can upregulate NOX4 and promote H_2_O_2_ production,^[^ ^8^ ^]^ which agrees with our research findings and provides the prerequisite for abundant intracellular ROS generation by THN. Next, the in vitro •OH production capacity of THN+RT was evaluated. Upon X‐ray irradiation, TN, HN, and THN‐mediated RT induced the generation of large amounts of •OH in cells (Figure 3D). Surprisingly, THN+RT and HN+RT still exhibited significant •OH fluorescence at 1 h after irradiation, whereas the other control groups showed negligible fluorescence signals (Figure 3E). The fluorescence intensity of •OH in the THN+RT group was higher than that in the HN+RT group. Furthermore, THN can effectively increase the level of superoxide anions in tumor cells after RT (Figure S11, Supporting Information). This indicates that both HN and THN showed a good ability to catalyze the production of •OH from H_2_O_2_, which could be attributed to the chemodynamic therapeutic effect of Hemin, generating ROS more sustainably. Next, TPEPy‐I in THN was able to deposit more X‐ray energy than HN, thus producing more ROS. After THN+RT treatment, 4T1 cells exhibited more DNA damage (Figure 3F,G) and inhibition of colony formation (Figure 3I). In addition, the disulfide bonds present in THN and the ROS generated by RT jointly downregulated the GSH content in tumor cells (Figure 3H). The survival fraction (SF) of 4T1 cells from different radiation doses was then studied (Figure 3J). As the radiation increased, the cell SF of the THN group decreased, with a sensitizer enhancement ratio (SER) value of 3.16, with ≈2 times higher than that of TN and HN groups, respectively. These findings demonstrated that the combination of TPEPy‐I and Hemin in the THN group exhibited the strongest RT sensitization effects. To simulate THN‐mediated deep tumor killing in vitro, we conducted RT and photodynamic therapy by blocking chicken breast meat as a biological barrier in cell culture dishes. As shown in Figure S12 (Supporting Information), Ce6, and THN almost lost their photodynamic anti‐tumor effects when a 3.5 cm chicken breast meat was used as a barrier, while THN‐mediated therapeutic effect was not affected. The experimental results suggest that THN‐sensitizing RT offers advantages over photodynamic therapy or pure RT for treating deep tumors.

### 2.4 Activation of Dendritic Cell (DC) Maturation and Macrophage Polarization by THN

**Figure 4:**
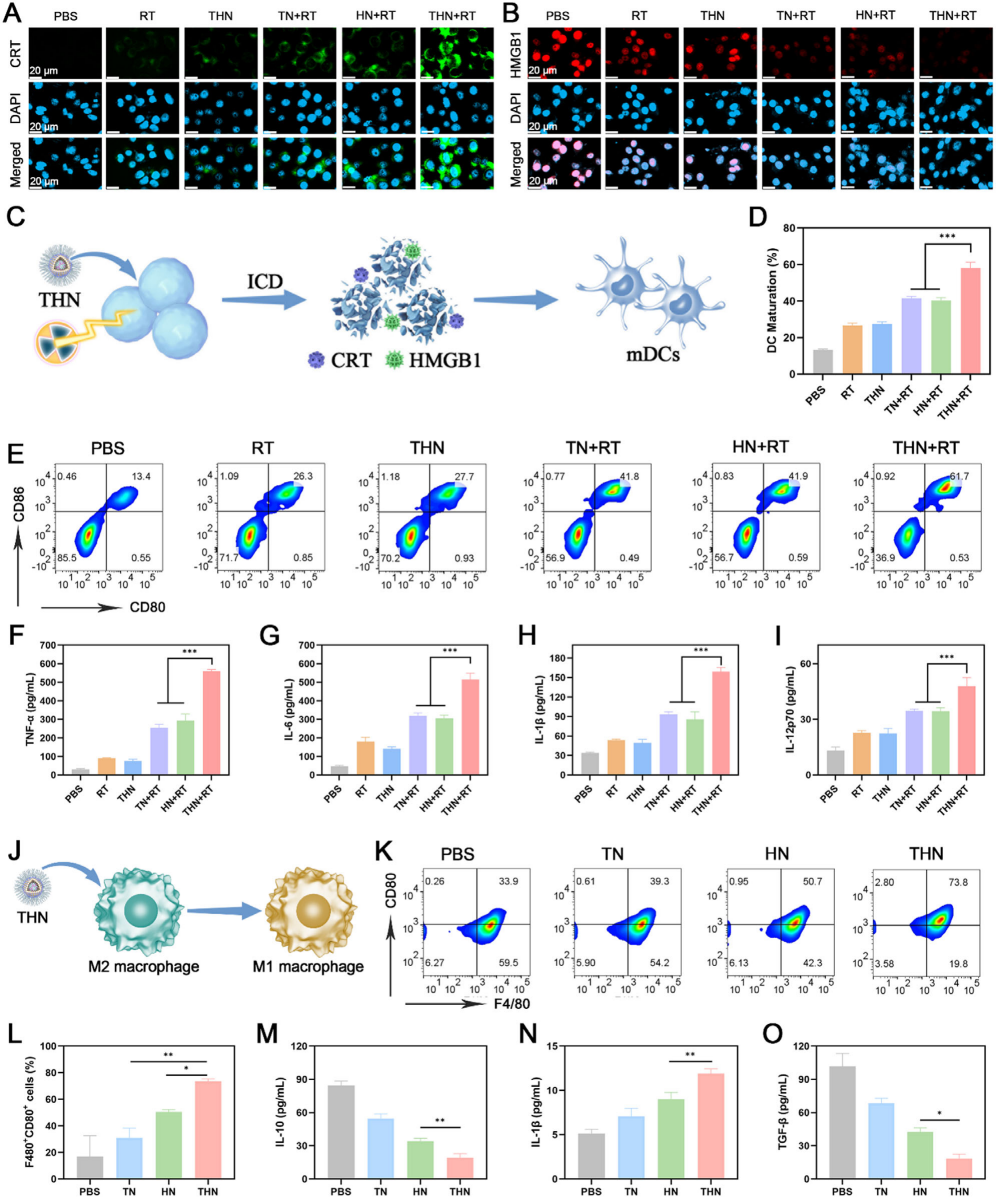
A) CLSM images and of CRT and B) HMGB1 expression in 4T1 cells upon different treatments. C) Schematic illustration of the maturation of BMDCs in vitro. D) Quantitative analysis and E) Flow cytometry analysis and of mature BMDCs after different treatments. Data are shown as the mean ± SD (*n* = 3). F) The levels of TNF‐α, G) IL‐6, H) IL‐1β and I) IL‐12p70 are secreted by matured BMDCs. J) Schematic illustration of the macrophage polarization in vitro. K) Flow cytometry analysis and L) Quantitative analysis of M1 type macrophages (F4/80^+^CD80^+^) after different treatments. M) The levels of IL‐10, N) IL‐1β, and O) TGF‐β are secreted by macrophages. TPEPy‐I concentration: 100 µg mL^−1^. RT:4Gy. Data are shown as the mean ± SD (*n* = 3). Statistical significance was calculated via one‐way ANOVA with Tukey's test: ns: Non‐Significant, ^***^ *p* < 0.001.

RT is widely regarded as one of the potent modalities for inducing ICDs in tumor cells.^[^ ^16^ ^]^ During RT, dying tumor cells release substantial amounts of damage‐associated molecular patterns (DAMPs) and tumor‐associated antigens, including calreticulin (CRT), which translocate from the endoplasmic reticulum to the cell membrane, and high mobility group box 1 (HMGB1), which is released from the nucleus into the extracellular environment.^[^ ^17^ ^]^ These species engage with specific receptors on DCs and macrophages, enhancing antigen presentation and activating specific antitumor immune responses.^[^ ^18^ ^]^ Despite numerous reports also demonstrating the efficacy of AIEgens in PDT‐based tumor treatment, these systems, activated via visible or NIR light, are limited by insufficient laser penetration depth, restricting their application against deep tumors.^[^ ^19^ ^]^ Moreover, the ICD induction relying on single phototherapy is constrained by the immunosuppressive tumor microenvironment, resulting in unsatisfactory antitumor immune activation. Our developed THN nanoparticles ingeniously leverage the radiosensitization properties of iodine‐containing AIEgens, which could be excited by X‐ray irradiation with much deeper penetration depth and elicit enhanced anti‐tumor immunity against deep tumors. More importantly, the immunostimulant capability of Hemin, as a ferroptosis inducer through Fenton reactions,^[^ ^20^ ^]^ could be synergistically enhanced with abundant ROS generation. Consequently, THN+RT is expected to induce ICD through multiple pathways, offering broader immunostimulatory potential than photodynamic‐immunotherapy approaches or traditional RT. CLSM results indicated considerably higher CRT expression (green fluorescence) in the THN+RT group than in other groups (**Figure** **4**A). HMGB1 expression in the nucleus was markedly reduced in these groups (Figure 4B). These results suggest that THN‐based nanoparticles enhance oxidative stress via RT and effectively induce ICD in tumor cells. Previous studies have shown that ferroptosis depends on lipid peroxidation caused by iron overload,^[^ ^21^ ^]^ and there are various ICD induction pathways, such as mitochondrial damage caused by reactive oxygen species burst, endoplasmic reticulum stress caused by radiotherapy and chemotherapy, etc.^[^ ^22^ ^]^ Tumor cells that undergo ferroptosis can also release DAMPs as adjuvants to enhance the tumor response. However, ferroptosis lacks sufficient evidence of the other two major characteristics of ICD: cytokine release and antigenic regulation.^[^ ^23^ ^]^ Therefore, THN combined with radiotherapy efficiently induces ICD to have better immune activation ability compared with a single ferroptosis inducer.

DC is a type of antigen‐presenting cell that plays a critical role in activating T cell‐mediated adaptive immune responses.^[^ ^16^, ^24^ ^]^ DAMPs released from tumor cells during ICD can serve as antigens to activate DCs and augment T cell‐mediated antitumor immunity.^[^ ^25^ ^]^ To determine the potential of THN‐induced ICD to stimulate DC maturation, 4T1 cells treated with THN+RT and bone marrow‐derived dendritic cells (BMDCs) were cocultured in a transwell system (Figure 4C). After 24 h of co‐culture, BMDCs were harvested and the expression levels of BMDCs maturation markers (CD86 and CD80) were detected. As depicted in Figure 4D,E, THN+RT‐treated tumor cells exhibited a substantially enhanced maturation rate (CD86^+^ CD80^+^) of BMDCs (61.7%) compared with untreated tumor controls (13.4%). To gain deeper insight into the functional status of BMDCs, the secretion levels of proinflammatory cytokines, including tumor necrosis factor (TNF)‐𝛼, interleukin (IL)‐1𝛽, IL‐6, and IL‐12p70, were quantified. As illustrated in Figure 4F–I, the secretions of TNF‐𝛼, IL‐1𝛽, IL‐6, and IL‐12p70 by BMDCs in the THN+RT group were considerably upregulated compared with those in the control group. These findings confirmed that THN+RT‐treated tumor cells could effectively activate the maturation of BMDCs.

Subsequently, the role of THN in macrophage activation was evaluated. Considering that tumor‐associated macrophages (TAMs) are mostly of the M2 phenotype,^[^ ^26^ ^]^ RAW264.7 macrophages were initially stimulated with IL‐4 and IL‐13 to obtain an M2 phenotype before being treated with THN (Figure 4J). Nuclear factor *𝜅*B (NF‐*𝜅*B) is an essential pathway of macrophages in responses, especially the inflammatory and immune responses to external stimuli.^[^ ^27^ ^]^ Hence, the activation of NF‐κB was examined using western blotting. The results showed that THN treatment induced intracellular NF‐κB activation (Figure S13, Supporting Information). Moreover, flow cytometry assays indicated that THN upregulated the expression of CD80 (M1 marker) (Figure 4K,L). Various cytokines secreted by macrophages play a vital role in regulating the immune response and suppressing cancer cell activity. As depicted in Figure 4M–O, THN‐treated macrophages considerably increased the secretion of the proinflammatory cytokine IL‐1𝛽 and suppressed the secretion of immunosuppressive cytokines transforming growth factor (TGF)‐𝛽 and IL‐10. This evidence indicated that THN could activate macrophage M1 polarization via the NF‐𝜅B signaling pathway.

### 2.5 In Vivo Antitumor Capacity of THN

**Figure 5:**
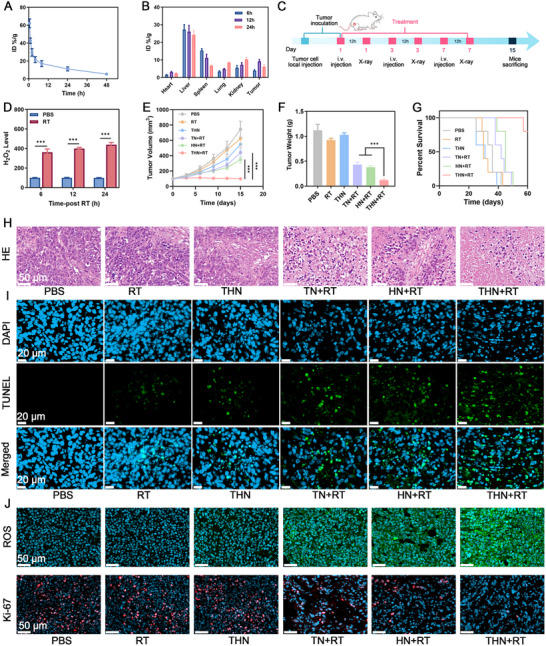
A) Pharmacokinetic curves of THN. B) In vivo biodistribution of THN after injections. C) Schematic diagram of therapy strategy. D) The H_2_O_2_ content in tumor tissue at different time points after RT. E) Tumor volumes were measured every 3 days across all groups. F) Tumor weights were recorded for each treatment at the end of the study. G) Survival curves after treatment. H) HE, I) TUNEL, J) ROS, and Ki‐67 staining of tumor sections after the indicated treatments. TPEPy‐I dose: 10 mg kg^−1^. RT dose: 4 Gy. Data are shown as the mean ± SD (*n* = 5). The p values were calculated via one‐way ANOVA, ^***^ *p* < 0.001.

Based on the finding that THN can effectively induce tumor cell ICD and macrophage M1 polarization in vitro, attempts were made to explore its tumor therapeutic effects in vivo and the corresponding antitumor immune response. A BALB/c mouse breast cancer model was established via the subcutaneous injection of 4T1 cells. The pharmacokinetics and biodistribution of THN were initially evaluated, as presented in **Figure** **5**A,B. A high accumulation of THN at the tumor site was observed 12 h after intravenous injection. As shown in Figure 5C, when the tumor volume reached 100 mm^3^, the tumor‐bearing mice were randomly divided into six groups of five mice each, as follows: 1) PBS, 2) RT (4 Gy), 3) THN, 4) TN+RT, 5) HN+RT, and 6) THN+RT. The H_2_O_2_ content in the tumor site after RT increased significantly, providing the source for the chemodynamic process of Hemin (Figure 5D). During the 15‐day observation, the tumors in the PBS group grew rapidly, whereas treatment with THN+RT considerably inhibited tumor growth with prolonged survival rates of 80% to 60 days (Figure 5E–G), which confirmed its antitumor therapeutic effect in vivo. The hematoxylin and eosin (H&E), DCFH‐DA, Ki‐67, and TdT‐mediated dUTP nick‐end labeling (TUNEL) staining of tumor tissues confirmed that THN+RT induced extensive tumor oxidative stress and cell death (Figure 5H–J). No significant weight loss was observed in the mice during the treatment (Figure S14, Supporting Information), and no abnormalities were detected in organs or biochemical indicators (Figures S15 and S16, Supporting Information), revealing excellent biosafety of the treatments.

**Figure 6:**
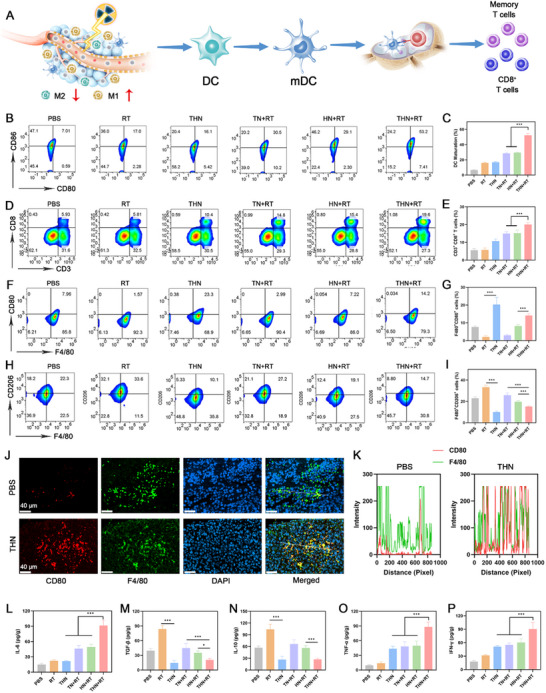
A) Regulation of immune cells by THN plus RT. B) Flow cytometry analysis of intratumoral DC maturation and C) quantitative analysis. D) Flow cytometry analysis of CD8^+^ T cells in spleens and E) quantitative analysis. F) Flow cytometry analysis of intratumoral M1‐type macrophages and G) quantitative analysis of M1‐type macrophages. H) Flow cytometry analysis of intratumoral M2‐type macrophages and I) quantitative analysis of M2‐type macrophages. J) CD80 (red) and F4/80 (green) immunofluorescence images of tumor sections after first treatment. K) Fluorescence intensity profile of white line in Figure 5J. L) ELISA measurements of intratumorally secretions of (L) IL‐6, M) TGF‐β, N) IL‐10, O) TNF‐𝛼, and (P) IFN‐𝛾 (*n* = 5). TPEPy‐I dose: 10 mg kg^−1^. RT dose: 4 Gy. The data are presented as the means ± SD (*n* = 5). The p values were calculated via one‐way ANOVA, ^*^ *p <* 0.05, and ^***^ *p <* 0.001.

To further examine the in vivo antitumor immune response induced by THN‐based ORS combined with RT and its impact on the immune microenvironment, an in‐depth analysis of DCs, TAMs, and CD8^+^ T cells of treated mice was conducted (**Figure** **6**A). Initially, the proportion of mature DCs in lymph nodes was assessed using a flow cytometer. The findings signified a substantial increase in the expressions of the DC costimulatory markers CD80/86 in the TN+RT and HN+RT groups, with the highest proportion observed in the THN+RT group at 53.2%, compared with a mere 7.01% in the PBS group (Figure 6B,C). Subsequently, the proportion of CD3^+^CD8^+^ T cells was evaluated. The TN+RT and HN+RT groups exhibited CD8^+^ T cell proportions of 14.8% and 15.4%, respectively, whereas the THN+RT group showed a significantly higher proportion of 19.6%, compared with only 5.93% in the PBS group (Figure 6D,E). Analysis of TAMs demonstrated a shift from immune‐suppressive M2‐type macrophages to immune‐active M1‐type macrophages in the THN+RT group. With the most pronounced alterations in the THN group, the proportion of M1‐type TAMs in the THN group increased from 7.96% to 23.3%, whereas that of M2‐type TAMs decreased from 22.3% to 10.1% (Figure 6F–I). These findings indicate that RT promotes M2 macrophage polarization while suppressing M1 macrophages, whereas THN effectively reverses this effect. Previous studies have reported that RT induces infiltration of M2 macrophages, resulting in tumor immune suppression and promoting tumor recurrence.^[^ ^28^ ^]^ Therefore, THN serves not only as a radiosensitizer but also as an immune regulator to further enhance the post‐RT immunotherapy effect, making it more clinically valuable than other simple radiosensitizers. The maturation of DCs and polarization of M1 macrophages can effectively activate cytotoxic T lymphocytes in tumors. The presence of M1 macrophages within the tumor tissue was verified using immunofluorescence staining for F4/80 (green, macrophage marker) and CD80 (red, M1 macrophage marker). Immunofluorescence results showed significantly intensified F4/80^+^ and CD80^+^ positive fluorescence signals after the THN treatment, which provided further evidence for the increased intratumoral infiltration of M1 macrophages (Figure 6J,K).

Various types of cytokines within the tumor microenvironment also play a key role in modulating the immune response. TGF‐𝛽 and IL‐10 inhibit the immune function of cytotoxic T cells in tumors and block the release of key proteins involved in the “cytotoxicity program,” such as perforin, granzymes, and cytotoxins.^[^ ^29^ ^]^ In contrast, TNF‐𝛼, IL‐6, and IFN‐𝛾 directly kill tumors or promote antitumor immune responses.^[^ ^30^ ^]^ To determine the changes in the tumor immune microenvironment, the major immunomodulatory cytokines in the tumor were quantified using enzyme‐linked immunosorbent assay (ELISA). As shown in Figure 6L–P, the levels of intratumoral TGF‐𝛽 and IL‐10, indicators of the immunosuppressive microenvironment, were considerably downregulated by the THN+RT treatment, whereas THN+RT treatment markedly upregulated the levels of the antitumor cytokines TNF‐𝛼, IL‐6, and IFN‐𝛾. These findings confirmed that the THN+RT treatment reversed the tumor immunosuppressive microenvironment and activated a systemic antitumor immune response.

**Figure 7:**
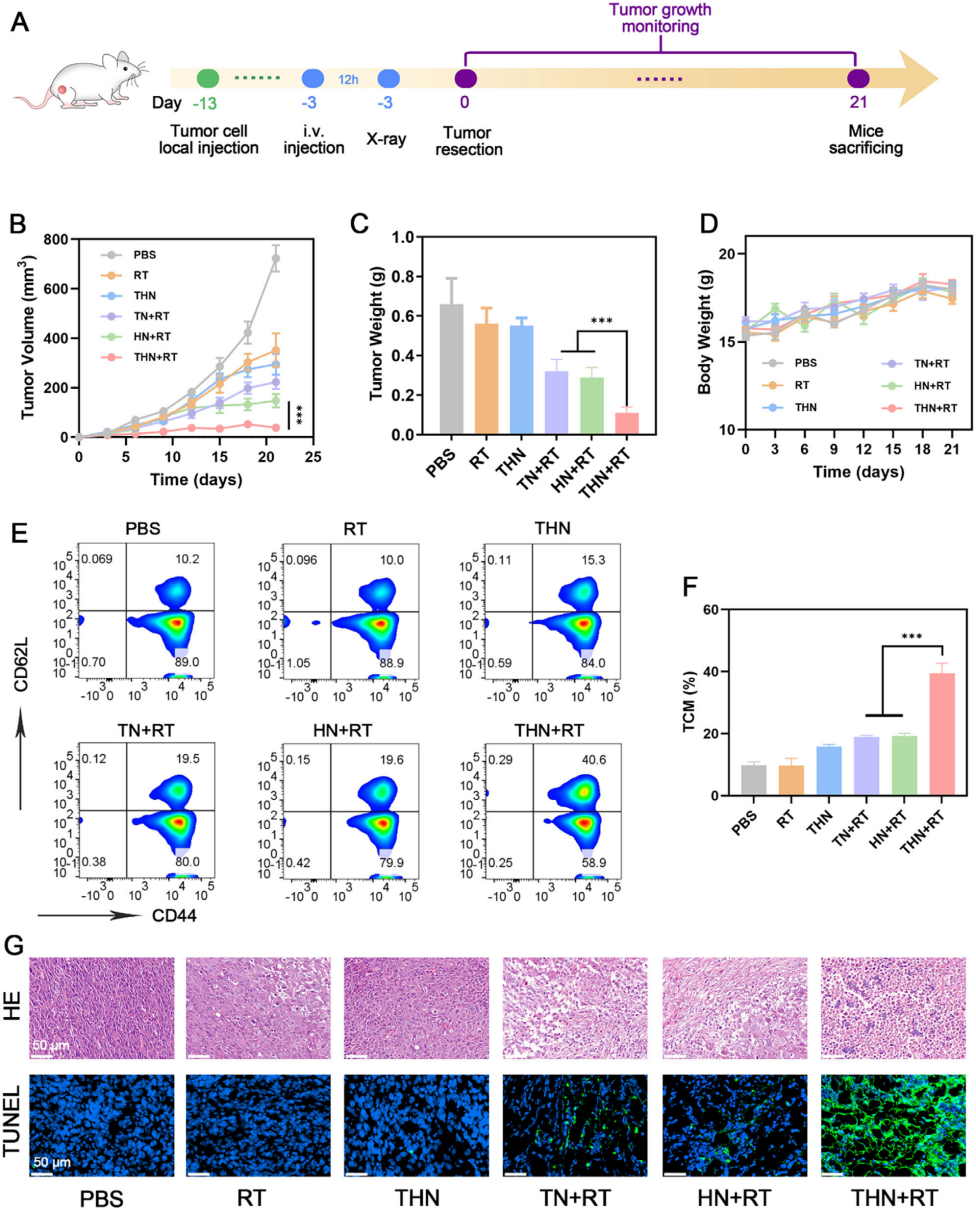
A) Schematic illustration of the studies of recurrence 4T1 model therapy. B) Evolution of the tumor volume and C) tumor weight after various treatments. D) Changes in body weight during the treatment period. E) Flow cytometry analysis and F) quantification of CD44^+^CD62L^+^ T lymphocytes in blood on the 7th day after the first treatment. G) H&E, and TUNEL staining of tumor sections after the indicated treatments. Data are shown as the mean ± SD (*n* = 5). Statistical significance was calculated via one‐way ANOVA with Tukey's test: ^***^ *p* < 0.001.

Given the effective activation of antitumor immunity and induction of tumor ICD by THN+RT, we hypothesized that it could also induce tumors to produce in situ tumor vaccines, activating the body's long‐term antitumor immune response. To validate the efficacy of THN+RT in preventing tumor recurrence, we established a postoperative recurrence model (**Figure** **7**A). The results showed that after THN+RT and surgical treatment, tumor recurrence in mice was significantly inhibited, whereas the control group mice exhibited varying degrees of tumor recurrence (Figure 7B,C; Figure S17, Supporting Information). No significant body weight loss was observed in mice during the treatment (Figure 7D). The underlying mechanism was subsequently investigated, which revealed that the proportion of central memory T cells (TCM) in the bloodstream of mice was substantially increased 1 week after THN+RT treatment (Figure 7E,F). TCM is a type of T cell with long‐term memory produced by naive T cells activated by antigens, which can nest in lymph nodes and receive antigen stimulation.^[^ ^31^ ^]^ Clinical studies have shown that TCM and cloned T cells derived from it are highly efficient antitumor immune T cells.^[^ ^32^ ^]^ After THN+RT treatment, the mice produced more T cells; furthermore, TCM increased significantly, which was the main reason for the considerable inhibition of tumor recurrence in the THN+RT group. Recently, there have been some reports on the promotion of tumor immunotherapy by X‐rays combined with nanomedicines.^[^ ^8^, ^33^ ^]^ However, researchers have paid less attention to the issues of persistent reactive oxygen species generation and anti‐tumor recurrence after radiotherapy. Cancer stem cells have a relatively high resistance to radiotherapy and can still induce recurrence after RT.^[^ ^34^ ^]^ The persistent anti‐tumor toxicity mediated by THN can effectively activate memory T cells and achieve long‐term tumor suppression. Tumor tissue slices were analyzed, which revealed morphological alterations and apoptosis in the THN+RT group, confirming the therapeutic effect of THN+RT on recurrent tumors (Figure 7G).

## 3 Conclusion

In summary, THN, a GSH‐responsive nanomedicine loaded with iodine‐containing AIE molecules and Hemin, was prepared as an ORS for X‐ray‐induced sustained ROS generation and efficient antitumor immunotherapy. Under external X‐ray irradiation, THN effectively deposited X‐rays and generated abundant ROS, which augments RT to induce cancer ICD. THN‐mediated radiosensitization enhanced tumor cells to produce H2O2, thus promoting the chemodynamic process of Hemin in THN and catalyzing H2O2 to •OH continuously. In addition, THN can polarize M2 macrophages into the M1 phenotype, alleviate the immunosuppressive microenvironment induced by tumor radiotherapy, and enhance the cytotoxic activity of CD8^+^ T cells. More importantly, THN‐sensitized RT can inhibit post‐surgical tumor recurrence and increase the proportion of memory T cells in the bloodstream. Therefore, THN serves not only as an ORS but also as a tumor immunomodulatory agent that enhances radioimmunotherapy, with enormous potential for clinical transformation. In future studies, we aim to optimize the tumor‐targeting capacities of THN and investigate its potential to improve the efficacy of immune checkpoint inhibitors.

## Conflict of Interest

The authors declare no conflicts of interest.

## Supporting information



Supporting Information

## Data Availability

Data is available from the corresponding author upon reasonable request. Source data are provided in this paper.
