# Supplementary material for: Organic AIE Nanoradiosensitizer Potentiates X‐Ray Triggered Continuous Reactive Oxygen Species Generation for Potent Cancer Radioimmunotherapy
Source: Adv Mater. 2025 Jun 19;37(35):2502898. doi: 10.1002/adma.202502898 (PMC12411993; doi:10.1002/adma.202502898)
Supplement: Supplementary file 1 — Supporting Information [file ADMA-37-2502898-s001.docx]

**Experimental Procedures**

**Materials**

1,2-Distearoyl-sn-glycero-3-phosphoethanolamine-disulfide bond-Polyethylene Glycol (DSPE-SS-PEG) was purchased from Shanghai Yare Biotech, Co., Ltd. (China). Ferriprotoporphyrin IX chloride (Hemin), Chlorin e6 (Ce6), DCFH-DA, HPF, SOSG, DHR123 and 3,3′,5,5′-Tetramethylbenzidine (TMB) were purchased from Sigma-Aldrich (USA). Lyso-Tracker Green, ELISA kit and cell culture medium used in this work were purchased from Guangzhou Ruiao Biotechnology Co., Ltd. (China). The Antibodies for Calreticulin (CRT) was purchased from Beyotime Company (China). Rhodamine-phalloidin and 4',6-diamidino-2-phenylindole (DAPI) were purchased from Shanghai Yeasen Biotech Co, Ltd. APC-anti-mouse CD3, PE-anti-mouse CD8a, FITC-anti-mouse CD4, APC-anti-mouse F4/80, FITC-anti-mouse CD86, FITC-anti-mouse CD206, APC-anti-mouse CD11c and PE-anti-mouse CD80 were purchased from Biolegend Corp. 3,3'-Dioctadecyloxacarbocyanine perchlorate (DiO) was purchased from Shanghai Macklin Biochemical Technology Co., Ltd. (China). All of the aqueous solutions were prepared using purified deionized (DI) water purified with a purification system (Direct-Q3, Millipore, USA). The other solvents used in this work were purchased from Sinopharm Chemical Reagent (China) and Shanghai Macklin Biochemical Technology Co., Ltd. (China).

**Preparation of TPEPy-I**

TPEPy-I was prepared according to the reported work^[1]^. Briefly, Compound 1 (0.100 mg, 0.2 mmol), 1,4-dimethylpyridine-1-iodide (0.50 mg, 0.22 mmol), 20 mL of absolute ethanol and a few drops of piperidine were added to a flask under N_2_ atmosphere. The reaction mixtures were stirred overnight at 80 °C. After cooling down the reaction, the solvent was removed under reduced pressure and the crude product was purified by column chromatography (dichloromethane: methanol = 20:1) to afford 0.13 g of dark red solid as TPEPy-I. UV-vis spectra and fluorescence spectra were measured on the HORIBA Duetta spectrometer by using a 1-cm cuvette at 25°C. The excitation wavelength for fluorescence spectroscopy detection is 488 nm. ^1^H and ^13^C nuclear magnetic resonance (NMR) spectra were measured on a Bruker AVANCE 400 MHz fully digital superconducting NMR spectrometer using DMSO-*d*6 as solvents, and tetramethylsilane (TMS; δ = 0 ppm) was chosen as the internal reference. High-resolution mass spectra (HRMS) were obtained on a Bruker ultrafleXtreme mass spectrometer system (Agilent 6500 Q-TOF LC/MS) operated in time-off-light (TOF) mode. For the NMR measurement, it is to dissolve 5mg TPEPy-I material in 0.5 mL of DMSO-d6 solvent and then add this solution to the nuclear magnetic resonance (NMR) tube with a dropper. ^1^H NMR (400 MHz, DMSO-*d6*) δ 8.81 (d, *J* = 6.8 Hz, 2H, 2ArH), 8.22 – 8.14 (m, 3H, 3ArH), 7.53 (d, *J* = 8.5 Hz, 2H, 2ArH), 7.24 – 7.11 (m, 5H, 5ArH), 7.01 (t, *J* = 7.9 Hz, 4H, 4ArH), 6.96 – 6.81 (m, 4H, 2ArH, C=CH_2_), 6.72 (dd, *J* = 13.8, 8.8 Hz, 4H, 2ArH, CH_2_=C), 4.23 (s, 3H, NCH_3_), 3.69 (d, *J* = 2.4 Hz, 9H, OCH_3_); ^13^C NMR (101 MHz, DMSO-*d6*) δ 157.92, 157.79, 152.07, 146.66, 144.84, 144.39, 140.55, 139.44, 137.94, 135.46, 133.59, 133.36, 132.07, 132.02, 131.66, 130.80, 130.53, 125.19, 124.98, 123.03, 121.69, 113.35, 113.16, 54.91, 46.77; HRMS (ESI): *m/z* [M-I]+ calcd. for C_41_H_36_NO_3_S, 622.2410, found: 622.2424.

**Preparation of TPEPy-I and Hemin loaded liposomes (THN), TPEPy-I loaded liposomes (TN), Hemin loaded liposomes (HN) and bare liposomes (BN)**

A mixture of TPEPy-I (1.3 μmol), Hemin (1.5 μmol), DSPE-SS-PEG2000 (2 μmol) and chloroform (1mL) was sonicated (12 W output) to obtain a clear solution. The mixture was quickly injected into 9 mL of water, which was sonicated vigorously in water for 2 min. The mixture was stirred in fume food for 12 h to remove the chloroform. Then the suspension was dialyzed against water (molecule weight cut off 100 kDa) for 24h. Finally, the THN was obtained by freeze-drying. The content of Hemin and TPEPy-I was determined by ICP-AES and fluorescence spectrometer, respectively. The TN were prepared in the same way, except for removing the Hemin. The HN was prepared in the same way, except for removing the TPEPy-I. The BN was prepared in the same way, except for removing the drugs. UV-vis spectra and fluorescence spectra were measured on the HORIBA Duetta spectrometer by using a 1-cm cuvette at 25°C. The excitation wavelength for fluorescence spectroscopy detection is 488 nm. The particle size was measured by DLS (Malvern Zetasizer Nano ZS90).

The morphology of synthesized materials was observed with field-emission TEM (JEM-F200). Specifically, at room temperature, PBS solution containing 50 μg/ml of nanomaterials was dropped onto the carbon support membrane carrier wire (Zhongjingkeyi (Beijing) Film Technology Co., Ltd). After standing for several minutes, the excess liquid was absorbed from the edge of the copper mesh using filter paper. Finally, the morphology was observed on the TEM. Furthermore, after 4 Gy X-ray irradiation of 50 μg/ml THN PBS solution, TEM detection was performed using the same method.

**Cell lines**

4T1 Cells were cultured in RPMI 1640 containing 10% fetal bovine serum (FBS), 2.5g/L glucose, 0.11 g/L Sodium Pyruvate, 100 U/mL penicillin G sodium and 100 mg/mL streptomycin sulfate (complete 1640 medium). RAW264.7 cells were grown in DMEM supplemented with 10% FBS, 100 U/mL penicillin G sodium and 100 mg/mL

streptomycin sulfate. Both 4T1 and RAW264.7 cells were maintained at 37℃ in a humidified and 5% CO_2_ incubator.

To obtain cell lysates, wash 5×10^6^ cells three times with 1×PBS (800g, 5 minutes). Cell lysates were obtained by hypotonic treatment with 20 mM tris-HCL, 10mM KCl, 2mM MgCl_2_ and protease inhibitors. Then, the cells were crushed 20 times with a homogenizer and centrifuged (3200g, 5min). The supernatant is the cell lysate.

**Animal tumor models**

Female BALB/c aged 5-6 week were purchased from Vital River Company (Beijing, China). Balb/c mice were subcutaneously injected with 5 × 10^6^ 4T1 cells into the right flank. The animal experiments were carried out according to the protocol approved by the Ministry of Health in People’s Republic of PR China and were approved by the Administrative Committee on Animal Research of the Guangxi Medical University.

**·OH detection**

84 μL of PBS (pH 7.4 or 6.5) containing 1 μL of TMB (20 mg/mL in DMSO) were treated with: (1) H_2_O_2_; (2) BN+ H_2_O_2_; (3) TN+ H_2_O_2_; (4) HN+ H_2_O_2_; (5) THN+ H_2_O_2_. The concentration of TPEPy-I was 20 μg/mL. The UV-vis absorbance spectra of oxidized TMB were recorded. The absorbance of 652 nm was measured by a microplate reader.

**ESR measurements**

ESR spectroscopy was employed using 5,5-dimethyl-1-pyrroline N-oxide (DMPO) as a spin-trapping agent for •OH. Dark EP tubes with 80 μL of 100 mM DMPO solution, then different mixed solution was added into: (1) 20 μL of H_2_O_2_ (10 mM, pH = 6) + THN (containing 20 μg/mL TPEPy-I) and (2) 20 μL of H_2_O_2_ (10 mM, pH = 6). Then, the mixture was transferred into a quartz capillary rapidly and detected by an ESR spectrometer (Bruker A300 EMX plus) at room temperature.

**X-ray induced reactive oxygen species detection**

DCFH-DA (DCF, 0.5 mL, 1 mM in ethanol) and aqueous NaOH solution (2 mL, 1.0 mM) were combined to create DCFH, which was then processed for 30 min at ambient temperature to obtain the stock solution of 200 μM. 20 μL DCFH (stock solution) was added to THN (in PBS), TPEPy-I (in DMSO/water mixture, *fw* = 99%), 10mM GSH pre-treated THN (in PBS, dialysis is used to remove excess GSH), TPEPy-I+Hemin (in DMSO), commercially available Ce6 (in DMSO/water mixture, *fw* = 99%) or PBS buffer solution to achieve a final concentration of 10 μM for DCFH, and photosensitizer concentrations of 50 μg/mL. Then the PL intensity of DCFH was recorded through HORIBA Duetta spectrometer after X-ray irradiation with different dosage in 96-well plates. The rate of general ROS production was determined by measuring the fluorescence intensity at 525 nm. Using HPF, SOSG and DHR123 with the same experimental procedure to measure the generation of hydroxyl radicals and superoxide anions by different materials under X-ray.

For time-dependent ROS generation detection, 20 μL DCFH (stock solution) was added to THN (in PBS), TPEPy-I (in DMSO/water mixture, *fw* = 99%), commercially available Ce6 (in DMSO/water mixture, *fw* = 99%) or PBS buffer solution to achieve a final concentration of 10 μM for DCFH, and photosensitizer concentrations of 50 μg/mL. Then the PL intensity of DCFH was recorded through HORIBA Duetta spectrometer after X-ray irradiation at different time points (The total dose of fixed radiotherapy is 4Gy, and the total irradiation time of this dose is approximately 3 minutes). The rate of general ROS production was determined by measuring the fluorescence intensity at 525 nm.

**Theoretical simulation method**

In this study, the Geant4 Monte Carlo toolkit (version G4.10.07) was used for microdosimetric particle transport simulations^[2]^. The Geant4-DNA extension was utilized to calculate energy deposition in water, while the Penelope Low Energy Package was applied to model interactions within nanoparticles^[3]^. To account for multiple scatterings of electrons and ions, the G4 Urban model was implemented with a 1 nm step cut. Additionally, atomic de-excitation processes, including fluorescence and Auger electron emission, were enabled in all simulations. Iodine had mass fractions of 5.4 wt% in THN, and the remaining elements consisted of carbon, hydrogen, oxygen, and so on. For distance-dependent simulations, the nanoparticles were placed at the center of a water medium, and the dose enhancement ratio (DER) was calculated under small-animal irradiation conditions (The X-ray was generated by a tungsten target with a peak energy of 100 keV, which was also simulated using Geant4) at varying distances. Meanwhile, for energy-dependent simulations, the physical radiosensitization effects of a single nanoparticle under different monoenergetic X-ray irradiations were investigated. These simulations primarily focused on the radiosensitization enhancement at a single energy level, providing further insights into the underlying physical radiosensitization mechanisms.

***In vitro* cancer cell internalization study**

4T1 cells were seeded in 24-well plates and cultured for 12 h. Then, 10 μL THN (containing 100 μg/mL TPEPy-I) was added to the medium. Then, the cells were incubated for 1h ,3h or 6h at 37 °C and 5% CO_2_ and washed with PBS three times. The cells were harvested, stained with DAPI and Lyso-Tracker Green and imaged by using a CLSM. The fluorescence intensity was measured by ImageJ software.

**Production of intracellular H_2_O_2_**

10^6^ 4T1 cells were incubated for PBS or exposed to RT radiation (4 Gy) after 2,6,12 or 24 hours. After another 12 hours of incubation, the H_2_O_2_ content was measured by employing a commercial colorimetric H_2_O_2_ detection kit (Beyotime Biotech Inc, S0038). The assay was carried out according to the manufacturer’s instructions. Specifically, collect the cells into a centrifuge tube by centrifugation (1000g at 4℃ for 5 minutes) and add the lysis buffer at a ratio of 0.2 mL of hydrogen peroxide detection lysis buffer (Beyotime Biotech Inc, S0038-3). Then, thoroughly homogenize to break and lyse the cells. Centrifuge at about 12,000g at 4℃ for 5 minutes, and take the supernatant for subsequent determination. Then dissolve the hydrogen peroxide detection reagent in an ice-water bath. Add 50 μL of sample to the detection hole. Then add 0.1mL of hydrogen peroxide detection reagent (Beyotime Biotech Inc, S0038-1) to each well. Gently shake to mix well. Let it stand at room temperature for 30 minutes. Then immediately use a microplate reader to measure the absorbance of the solution at 560 nm. Finally, the concentration of hydrogen peroxide in the sample was calculated based on the standard curve (obtained from the detection of hydrogen peroxide standard substances (Beyotime Biotech Inc, S0038-2) of different concentrations).

**ROS generation detection in RAW 264.7 cells**

RAW 264.7 cells (8 × 10^4^ per plate) were incubated with different concentration of THN for 6h. Then, an ROS detection kit was added for total ROS detection by a confocal laser scanning microscope (CLSM; IX81, Olympus, Japan).

**·OH generation and NOX4 expression detection *in vitro***

4T1 cells (8 × 10^4^ per plate) were incubated with six different groups: (1) PBS; (2) RT (4Gy); (3) THN; (4) TN+RT; (5) HN +RT;(6) THN+RT. The TPEPy-I concentration was 100 μg/mL. Then, cells in groups 2, 4 and 6 were exposed to RT radiation (4 Gy) after 6 hours of different treatment. Then, an HPF detection kit was added for ·OH detection at different time points. The fluorescence intensity was measured by Flow cytometry. The expression of NOX4 was observed by immunofluorescence in cells of Group 1 and Group 2. Then, cells were detected by a confocal laser scanning microscope (CLSM; IX81, Olympus, Japan).

**DNA Double-Strand Breaks (γ-H_2_AX Immunofluorescence Analysis) *in Vitro***

4T1 cells (1.5 × 10^5^ per well) were seeded in a 12-well plate for 24 h. Then the cells were incubated 6 different groups: (1) PBS; (2) RT (4Gy); (3) THN; (4) TN+RT; (5) HN +RT;(6) THN+RT. The TPEPy-I concentration was 100 μg/mL. Then, cells in groups 2, 4 ,5 and 6 were exposed to RT radiation (4 Gy) after 6 hours of different treatment. Then the cells were fixed by 4% paraformaldehyde for 30 min, rinsed three times with PBS, treated with triton-X 100 for 10 min at room temperature to enhance cell permeabilization, and then rinsed three times with PBS again. The cells were exposed to a blocking buffer (1% BSA in PBS) for 1 h at room temperature and incubated with γ-H_2_AX antibody (dilution 1:500) overnight at 4 ºC. The next day, the cells were washed three times with PBS to remove the excess antibody and incubated with goat anti-mouse IgG (dilution 1:1000) for 1h at room temperature. Cells nuclei were stained by DAPI at room temperature. At last, the cells were imaged by confocal microscopy (Leica, Wetzlar, Germany). The fluorescence intensity was measured by ImageJ software.

**Detection of Intracellular GSH.**

The commercially available GSH assay kit was used to detect the depletion of GSH. 4T1 cells were incubated with 6 different groups: (1) PBS; (2) RT (4Gy); (3) THN; (4) TN+RT; (5) HN +RT;(6) THN+RT. The TPEPy-I concentration was 100 μg/mL. Then, cells in groups 2, 4 ,5 and 6 were exposed to RT radiation (4 Gy) after 6 hours of different treatment. After another 1 hour of incubation, the GSH content was measured by employing a commercial colorimetric GSH assay kit (Beyotime Biotech Inc, S0053). The assay was carried out according to the manufacturer’s instructions.

Specifically, wash the cells once with PBS, centrifuge to collect the cells, and carefully aspirate the supernatant. Add three times the volume of protein removal reagent M solution (Beyotime Biotech Inc, S0053-5) relative to the cell pellet volume, and vortex thoroughly to ensure complete mixing. Subject the samples to two rapid freeze-thaw cycles using liquid nitrogen and a 37°C water bath. Incubate the samples at 4°C or on ice for 5 minutes. Centrifuge at 10,000g for 10 minutes at 4°C. Collect the supernatant for total glutathione determination.

Take some of the samples prepared above for the total glutathione content to be tested and add the diluted GSH clearance auxiliary solution (Beyotime Biotech Inc, S0053-8 and S0053-9) at a ratio of 20 μL of diluted GSH clearance auxiliary solution for every 100 μL of sample. Immediately vortex and mix well. Then add the GSH removal reagent working solution at a ratio of 4 microliters for every 100 microliters of sample, immediately vortex and mix well, and react at 25 °C for 60 minutes. Using a 96-well plate, add the sample and 150 μL of the total glutathione detection working solution (Beyotime Biotech Inc, S0053-1, S0053-2, S0053-4 and S0053-7) , mix well and incubate at 25℃ for 5 minutes. Add 50 μL of 0.5mg/ml NADPH solution (Beyotime Biotech Inc, S0053-6) and mix well. Then immediately use a microplate reader to measure the absorbance of the solution at 412 nm. Finally, the concentration of GSH in the sample was calculated based on the standard curve (obtained from the detection of GSH standard substances (Beyotime Biotech Inc, S0053-3) of different concentrations).

**Clonogenic survival assay**

The effect of the radiosensitivity of 4T1 cells was assessed by a clonogenic assay. 500 cells per flask were seeded in 25 cm^2^ flasks and cultured in normoxia for 24 h. Flasks were treated under following conditions:(1) PBS; (2) RT (4Gy); (3) THN; (4) TN+RT; (5) HN +RT;(6) THN+RT. The TPEPy-I concentration was 100 μg/mL. Then, cells in groups 2, 4 ,5 and 6 were exposed to RT radiation (4 Gy) after 6 hours of different treatment. To allow formation of colonies, after radiation, the cells were then incubated in for another 10 days, without changing the media. To determine the clonogenic survival rate, cultures were first fixed with paraformaldehyde and then stained with trypan blue. Colonies with greater than 50 cells were counted under the microscope, and the survival fractions (SF) were calculated using the formula SF = colonies counted/cells seeded.

The effect of the radiosensitivity of 4T1 cells was assessed by a clonogenic assay. 500 cells per flask were seeded in 25 cm^2^ flasks and cultured in normoxia for 24 hours. Flasks were treated under following conditions: (1) PBS; (2) TN; (3) HN; (4) THN. The TPEPy-I concentration was 100 μg/mL. The cells were washed with PBS, and then exposed to 0, 2, 4, 6, or 8 Gy of radiation in sealed flasks containing 5 mL of complete medium after 6 hours of different treatment. To allow formation of colonies, after radiation, the cells were then incubated in for another 10 days, without changing the media. To determine the clonogenic survival rate, cultures were first fixed with paraformaldehyde and then stained with trypan blue. Colonies with greater than 50 cells were counted under the microscope, and the survival fractions (SF) were calculated using the formula SF = colonies counted/cells seeded.

**Cell toxicity assessment**

Typically, 4T1 cells were incubated in plates at 37 °C with 5% CO_2_ for 24 h; afterward, the culture medium was replaced by new culture medium, cells were incubated with 6 different groups: (1) PBS; (2) PBS+RT; (3) Ce6+L (white light laser, 0.1W/cm^2^, 10 min); (4) Ce6+RT; (5) THN+L and (6) THN+RT. After incubation for another 6 h, the viability of 4T1 cells was determined by a CCK-8 cell cytotoxicity assay according to the instructions. In addition, to demonstrate the advantages of X-rays over lasers, we purchased chicken breast meat (Guangzhou Yiyi Trading Co., LTD) and cut the chicken breast into slices with a thickness of 0.8 to 1cm with scissors and knife. Then, we stacked four pieces of chicken breast slices (the total thickness is approximately 3.5 cm) on the perforated plate to conduct the above CCK-8 experiment.

**Induction of immunologic cell death (ICD)**

After seeded in 48-well plate (2 × 10^4^ cells per well) for 12 h, 4T1 cells were pre-incubated incubated and treated with 6 different groups: (1) PBS; (2) RT (4Gy); (3) THN; (4) TN+RT; (5) HN +RT;(6) THN+RT. The TPEPy-I concentration was 100 μg/mL. Then, cells in groups 2, 4 ,5 and 6 were exposed to RT radiation (4 Gy) after 6 hours of different treatment. After another 12 hours, The cells were then washed with PBS three times, fixed with 4% PFA and permeabilized with 0.1% Triton X-100 for 10 min. After washed with PBS three times, the cells were blocked with 10% FBS, and incubated with anti-CRT antibody (Alexa Fluor® 647) or anti-HMGB1 antibody for 30 min. The cells were washed with PBS three times, then stained with DAPI for 20 min. Finally, the cells were washed with PBS three times and observed using CLSM. The fluorescence intensity was measured by Flow cytometry.

***In vitro* marrow-derived dendritic cells (BMDCs) maturation study**

BMDCs were isolated from 8-week-old Balb/c mice bone marrow. For BMDCs maturation assay, 1 × 10^5^ 4T1 cells were treated with 6 different groups: (1) PBS; (2) RT (4Gy); (3) THN; (4) TN+RT; (5) HN +RT;(6) THN+RT. The TPEPy-I concentration was 50 μg/mL. Then, cells in groups 2, 4 ,5 and 6 were exposed to RT radiation (4 Gy) after 6 hours of different treatment. Then, the treated 4T1 cells were cocultured with 1 × 10^6^ BMDCs. Then BMDCs were stained with anti-CD11c, anti-CD80, and anti-CD86 (Biolegend). Finally, the cells were sorted using flow cytometer (Beckman-Coulter, USA). The secretion levels of cytokines including TNF-α, IL-6, IL-1β and IL-12p70 in the samples were tested with ELISA kits (Guangzhou Ruiao Biotechnology Co., Ltd., China).

**Macrophages repolarization assay**

5 × 10^4^ RAW264.7 cells dispersed in 500 μL culture medium were seeded into per well of 24-well plates overnight, followed by incubation with IL-4 (40 ng mL−1) for 24 h to be polarized to M2 phenotype. Then 5 × 10^4^ M2 macrophages in 500 μL medium were cultured treated with 4 different groups: (1) PBS; (2) TN; (3) HN; (4) THN. The TPEPy-I concentration was 100 μg/mL. Then, the cells incubated with anti-mouse F4/80 antibody (BD biosciences, USA) and PE-Cy7 Rat anti-mouse CD86(BD biosciences, USA) for 30 min at 4 °C, followed by fixation and permeabilization. Anti-mouse CD206 (BD biosciences, USA) was then used for intracellular staining. All samples were resuspended by stain buffer for flow cytometry analysis (Beckman Cytoflex, USA). Flow cytometry data was analyzed with FlowJo software (version 10, BD Bioscience). The secretion levels of cytokines including IL-10, IL-1β and TGF-β in the samples were tested with ELISA kits (Guangzhou Ruiao Biotechnology Co., Ltd., China). The expression of NF-𝜅B in macrophages was determined by WB assay.

***In vivo* bio-distribution and pharmacokinetics study**

Balb/c mice (n = 3) received an intravenous injection of 100 μL PBS containing THN (with a TPEPy-I dose of 10 mg/kg). At various time points after the injection (i.e., 0.5, 1, 2, 4, 8, 24, and 48 h), 20 μL blood plasma was collected from the tail veins. The Hemin content in the blood was analyzed using High performance liquid chromatography (HPLC). Briefly, Blood samples were freeze-dried and extracted with 25 mL tetrahydrofuran (Ultrasonically extracted for 10 to 15 minutes). Then the samples were homogenized, and centrifugation at 1×10^4^ rpm for 10 min. The supernatants were diluted with methanol and separated on Agilent ZORBAX Eclipse. XDB. C8 (4.6 mm×150 mm), with 1% acetic acid and tetrahydrofuran (0.1% acetic acid: tetrahydrofuran=65:35) as mobile phase by gradient elution of 1 mL/min. Column temperature: 30 ℃. Detection wavelength: 399 nm.

Tumor bearing mice (n = 3) received an intravenous injection (i.v.) of 100 μL PBS containing THN (with a TPEPy-I dose of 10 mg/kg). At various time points after the injection (i.e.6, 12 and 24 h), major organs were collected from the mice and rinsed with PBS, and then freeze-dried. The weighed tissues were mixed with tetrahydrofuran, homogenized, and centrifugation at 1×10^4^ rpm for 10 min. The Hemin content in the supernatants was analyzed using HPLC as mentioned above.

***In vivo* antitumor study**

Female BALB/c aged 5-6 week were purchased from Vital River Company (Beijing, China). Balb/c mice were injected with PBS or exposed to RT radiation (4 Gy) after 6,12 or 24 hours. Then the mice were sacrificed to collect the tumor tissue. The H_2_O_2_ content was measured by employing a commercial colorimetric H_2_O_2_ detection kit (Beyotime Biotech Inc, S0038).

Female BALB/c aged 5-6 week were purchased from Vital River Company (Beijing, China). Balb/c mice were subcutaneous injected with 1 × 10^6^ 4T1 cells. When the tumor volume reaches 100 mm^3^ ,the mice were divided randomly into 6 different groups (Each group included 5 mice): (1) PBS; (2) RT (4Gy); (3) THN; (4) TN+RT; (5) HN +RT;(6) THN+RT. The TPEPy-I concentration was 10 mg/kg. Then, mice in group 2, 4 ,5 and 6 were exposed to RT radiation (4 Gy) after 12 hours of different treatment. The treatment was conducted on the 1,3 and 7 days. Mice body weight and tumor volume in all groups were monitored every 3 days. A caliper was employed to measure the tumor length and tumor width, and the tumor volume was calculated according to following formula. Tumor volume = tumor length × tumor width^2^ / 2. After 15 days of treatment, mice were sacrificed. The blood samples from these mice (≈1 mL) were collected for blood biochemistry analysis. The major organs including heart, liver, spleen, lung, and kidney were harvested, fixed in 4% of formalin, embedded in paraffin, sectioned into 4 μm slices, stained with hematoxylin and eosin (H&E), and observed by an optical microscope (BX51, Olympus, Japan). And the tumor tissues were weighed, and fixed in 4% neutral buffered formalin, processed routinely into paraffin, and sectioned at 4 μm. Then the tumor sections were stained with hematoxylin-eosin (H&E) staining, terminal -deoxynucleotidyl transferase mediated nick end labeling (TUNEL), anti-Ki-67 antibodies, anti-F4/80 and anti-CD 80 antibodies, and DCFH-DA and finally examined by using fluorescence microscope (IX81, Olympus, Japan). The fluorescence intensity was measured by Zen software.

For flow cytometric analysis, collected tumors, lymph nodes and spleens were ground using a 200-mesh filter to prepare corresponding single cells suspensions. The frequency of DC maturation in the lymph nodes was then examined by CD11c^+^ cell sorting kit (Thermo Fisher Scientific, USA) and flow cytometry after immunofluorescence staining with FITC-anti-CD80 and PE-anti-CD86 (Abcam)**.** The frequency of CD8+ T cells in the spleens was then examined by CD45^+^ cell sorting kit (Thermo Fisher Scientific, USA) and flow cytometry after immunofluorescence staining with FITC-anti-CD3 and PE-anti-CD8 (Abcam)**.** The frequency of M1 and M2 macrophages in the tumors was examined by CD11b^+^ cell sorting kit (Thermo Fisher Scientific, USA) and flow cytometry after immunofluorescence staining with FITC-anti-CD80 and PE-anti-F4/80 (Elabscience Biotechnology Co.,Ltd.) for M1 macrophages, FITC-anti-CD206 and PE-anti-F4/80 (Elabscience Biotechnology Co.,Ltd.) for M2 macrophages. The content of cytokines in tumors including IL-6, TGF-β, IL-10, TNF-α and IFN-γ were then analyzed with ELISA kits (Guangzhou Ruiao Biotechnology Co., Ltd., China) according to the manufacturer's instructions.

**Preventing tumor occurrence *in vivo***

Female Balb/c aged 5-6 week were purchased from Vital River Company (Beijing, China). Balb/c mice were subcutaneous injected with 1 × 10^6^ 4T1 cells on day -13.The mice were treated with 6 different groups (Each group included 5 mice): (1) PBS; (2) RT (4Gy); (3) THN; (4) TN+RT; (5) HN +RT;(6) THN+RT. The TPEPy-I dose was 10 mg/kg. Then, mice in group 2, 4 ,5 and 6 were exposed to RT radiation (4 Gy) after 12 hours of different treatment. The treatment was conducted on -3 day.Then, the primary tumors were resected on day 0. Mice body weight and tumor volume in all groups were monitored every 3 days. A caliper was employed to measure the tumor length and tumor width, and the tumor volume was calculated according to following formula. Tumor volume = tumor length × tumor width^2^/ 2. After 21 days of monitoring, mice were sacrificed. To study the central memory T cells (T_cm_) content in serum, the blood samples were collected on day 14 after the first treatment for immune memory study. The live CD8^+^ T lymphocytes were isolated by using the Mouse CD8^+^ T Cell Isolation Kit (Thermo Fisher Scientific, USA), stained with FITC-anti-CD62L (Biolegend, USA) and PE-anti-CD44 (Biolegend, USA). The subpopulations of T cells were finally analyzed on a flow cytometer.The tumor tissues were weighed, and fixed in 4% neutral buffered formalin, processed routinely into paraffin, and sectioned at 4 μm. The tumor sections were stained H&E staining, TUNEL and finally examined by using fluorescence microscope (IX81, Olympus, Japan).

**Statistical analysis**

Data analyses were conducted using the GraphPad Prism 5.0 software. For variance analysis, One-way analysis of variance (ANOVA) with Tukey’s post hoc test was used. p values of <0.05 were considered significant. *p < 0.05, **p < 0.01, ***p < 0.001.

**Supplementary figures**

**
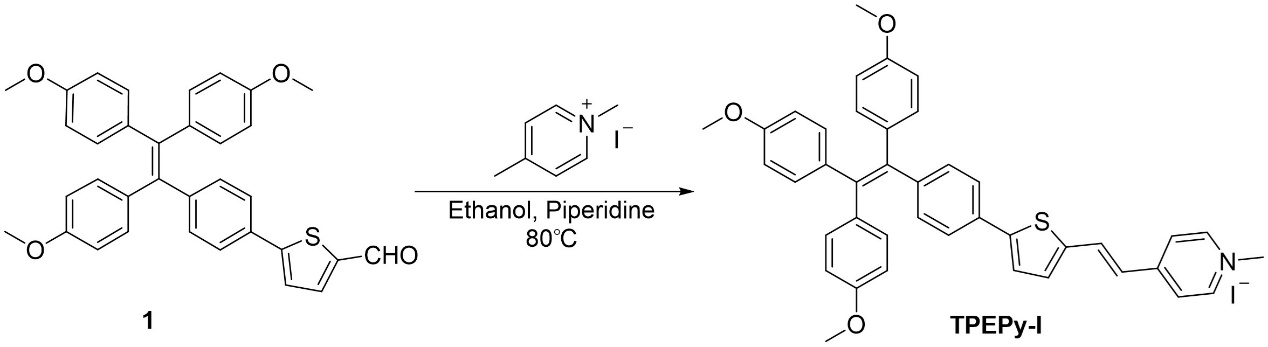
**

**Scheme S1.** The synthetic routes of TPEPy-I.


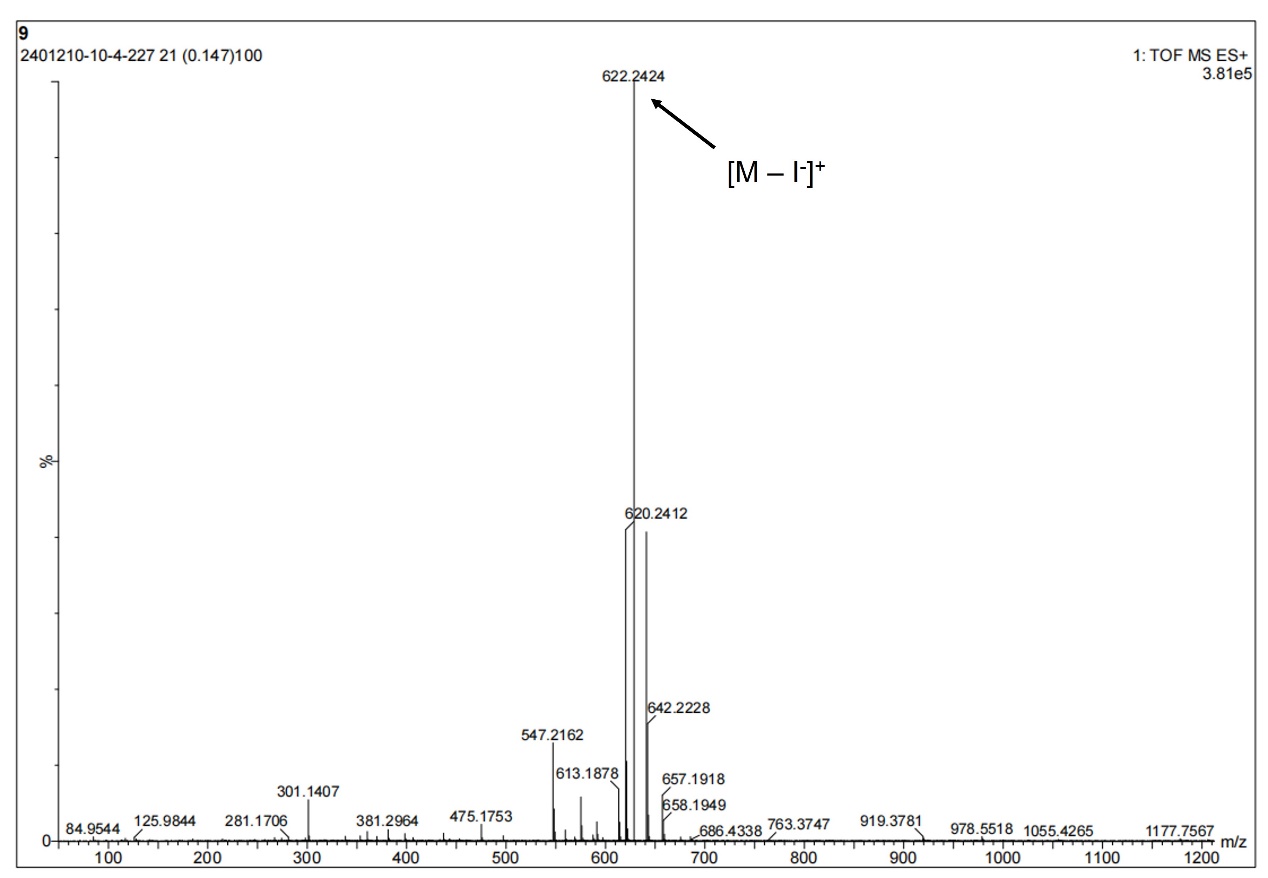


**Figure S1.** High Resolution Mass Spectrometry (HRMS) of TPEPy-I.

**
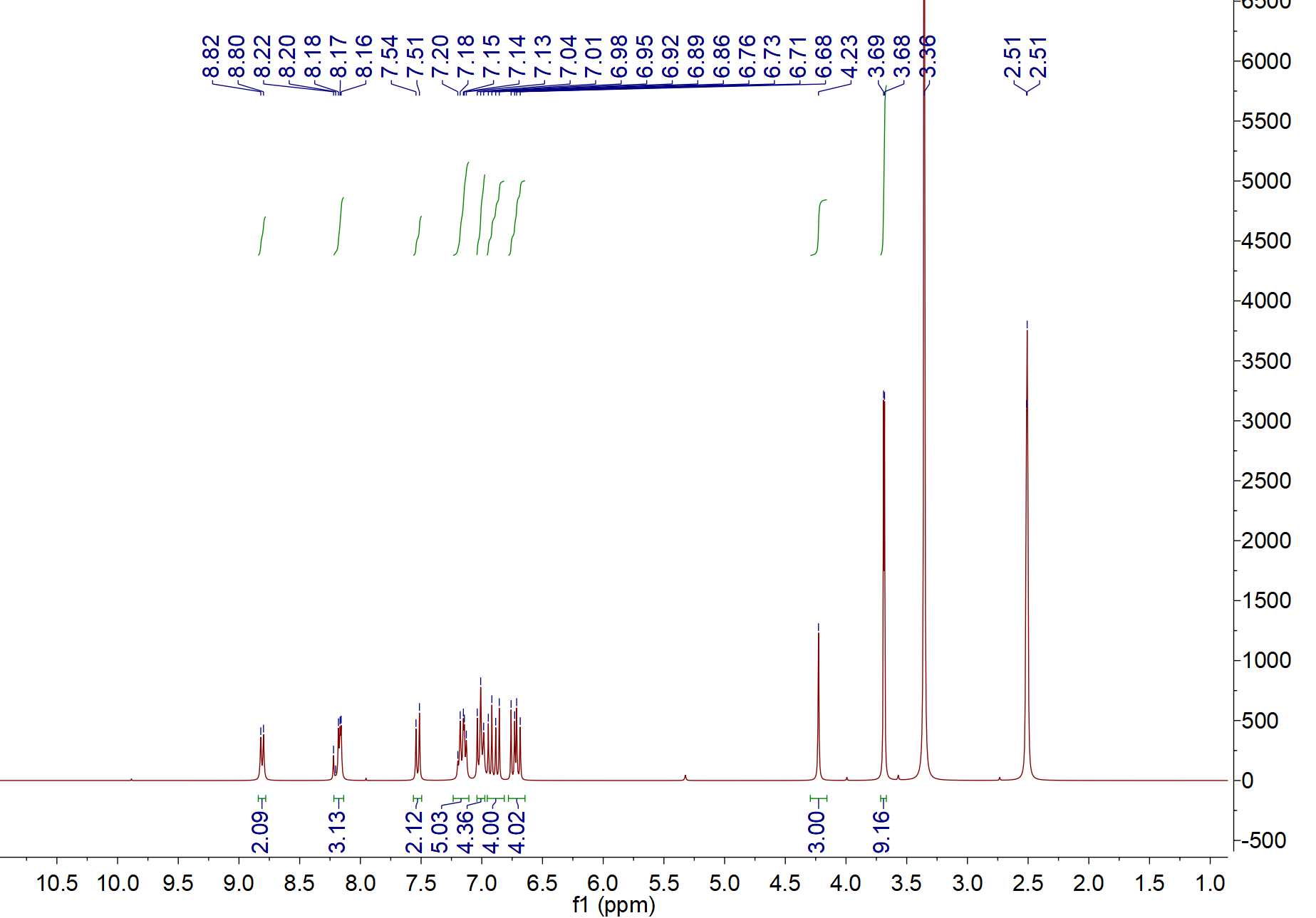
**

**Figure S2.** ^1^H NMR spectrum of TPEPy-I.


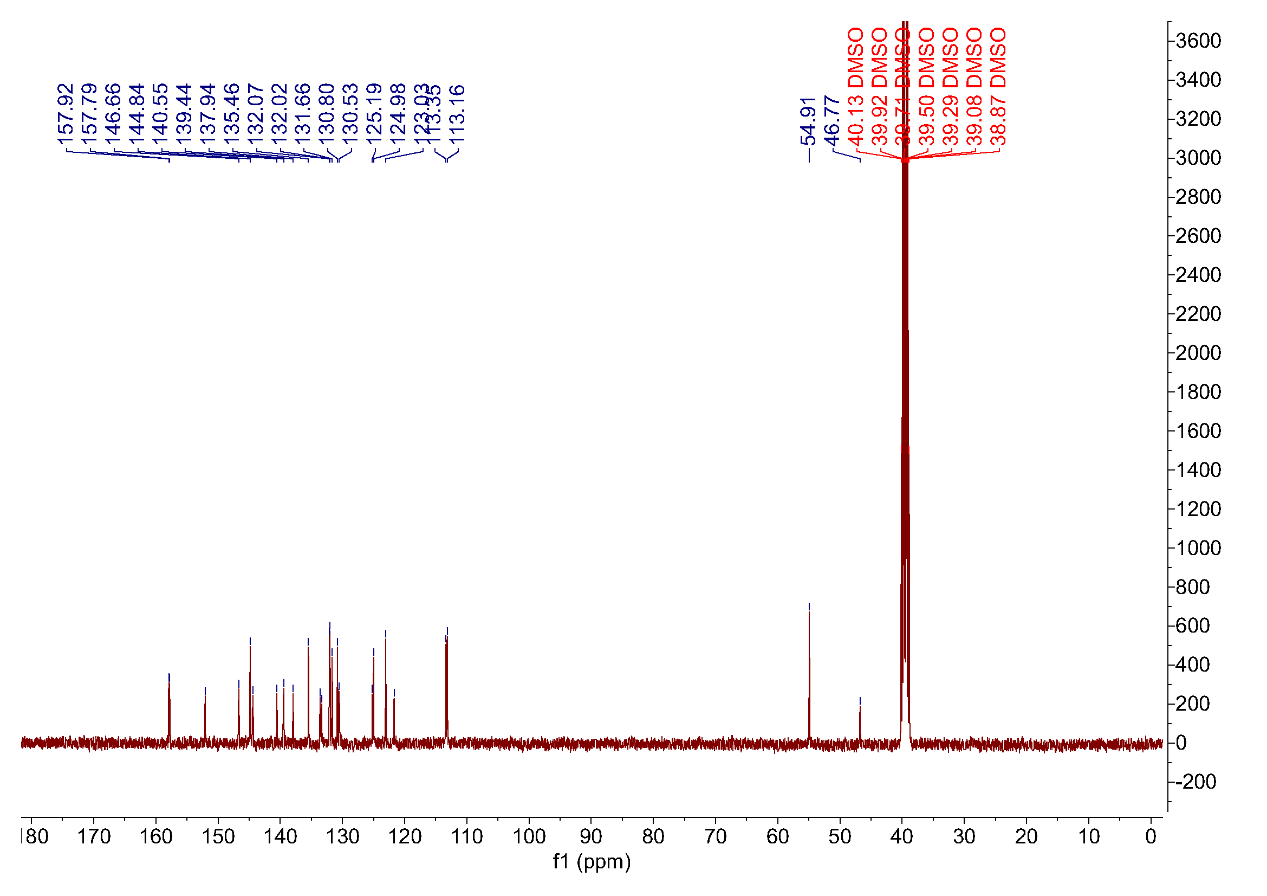


**Figure S3.** ^13^C NMR spectrum of TPEPy-I.


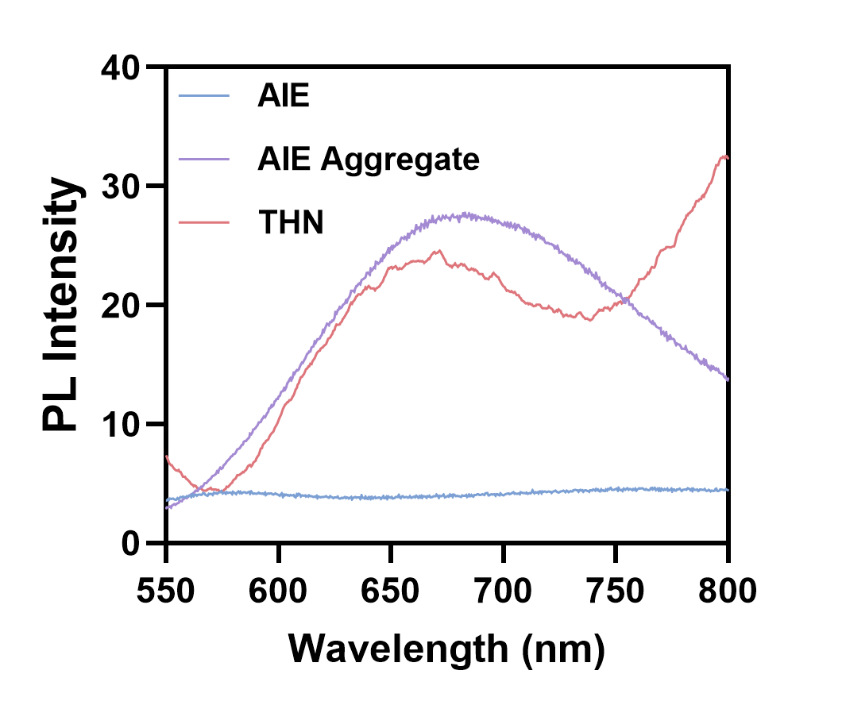


**Figure S4.** Photoluminescence (PL) spectroscopy of TPEPy-I as the AIE molecule in DMSO solution, AIE Aggregate (TPEPy-I in 99% water fraction) and THN in PBS solution. Excitation wavelength: 488 nm. Concentration of TPEPy-I: 10 μg/mL.


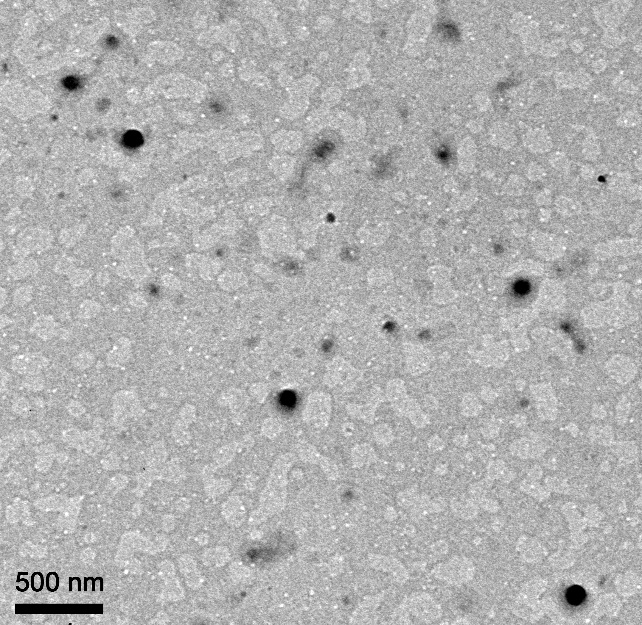


**Figure S5.** TEM image of THN after X-ray (4 Gy) irradiation. Concentration of TPEPy-I: 10 μg/mL.


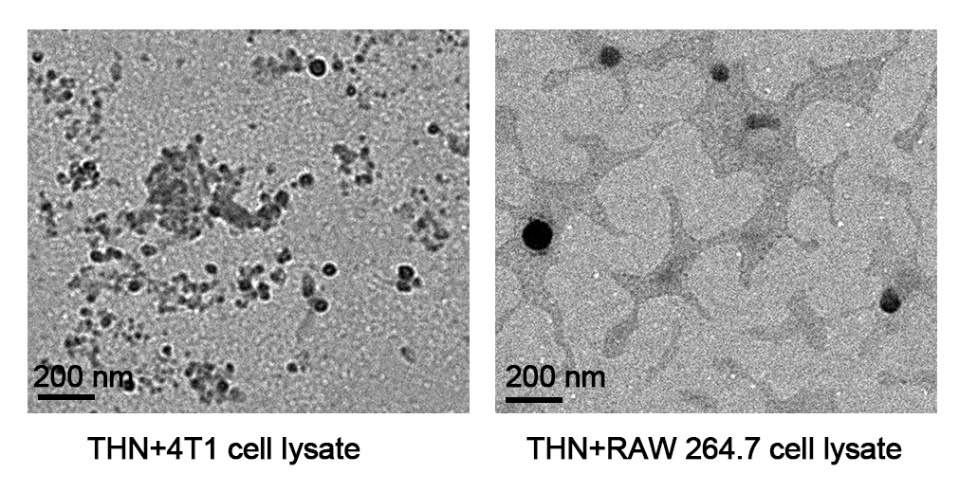


**Figure S6.** TEM image of THN after incubated with different cell lysate for 4h. Concentration of TPEPy-I: 10 μg/mL.


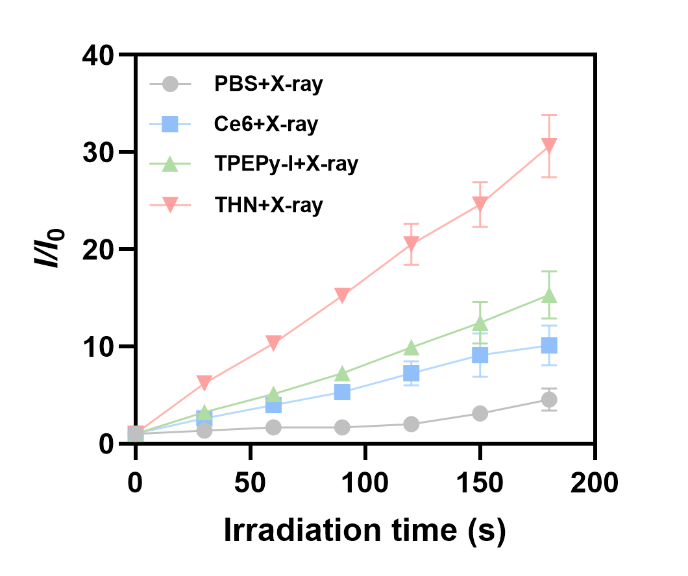


**Figure S7.** Total ROS generation with fluorescence enhancement of DCFH for different formulations upon X-ray irradiation with different time. Concentration of TPEPy-I and Ce6 are 50 μg/mL.


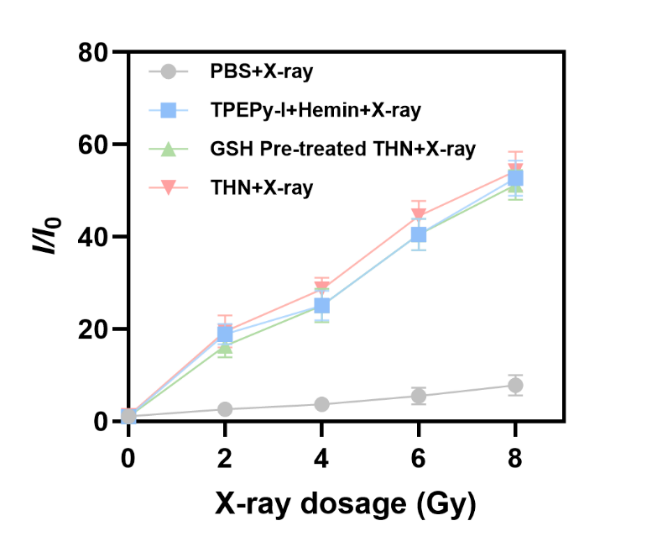


**Figure S8.** Total ROS generation with fluorescence enhancement of DCFH for TPEPy-I+Hemin (in DMSO), GSH Pre-treated THN (in water), and THN (in water) upon X-ray irradiation with different dosages.


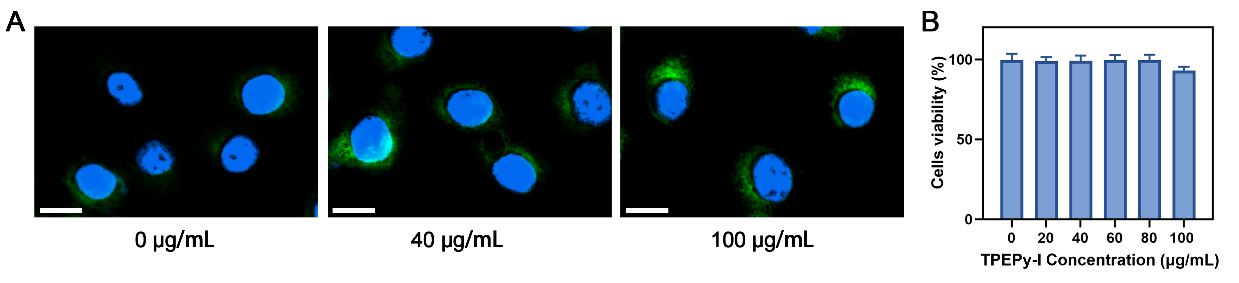


**Figure S9.** (A) CLSM images of ROS (DCFH-DA, green fluorescence) generated in RAW 264.7 cells upon THN treatments with different TPEPy-I concentration. Scale bars: 10 μm. (B) The effect of THN on the survival rate of RAW 264.7 cells with different TPEPy-I concentration. Data are shown as the mean ± SD (n = 3).


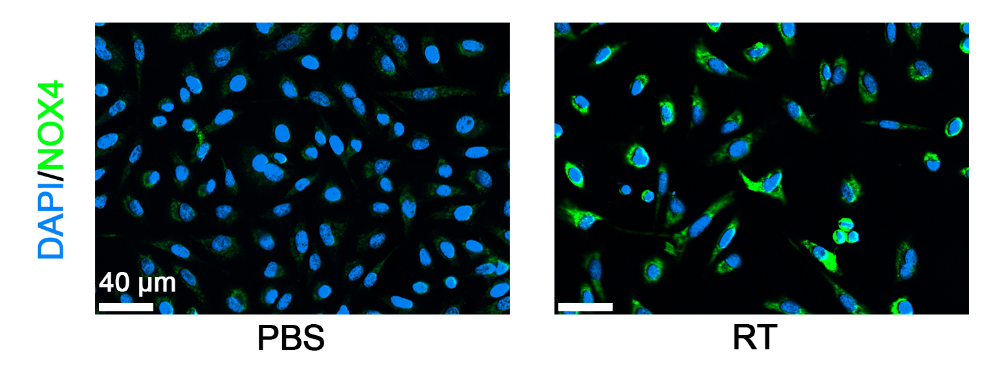


**Figure S10.** NOX4 staining of 4T1 cells after the indicated treatments. RT dose: 4 Gy.


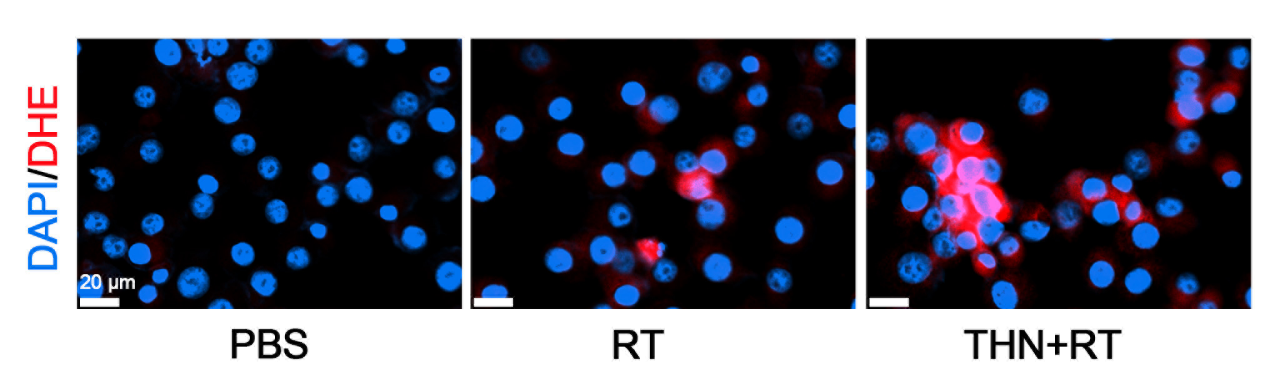


**Figure S11.** Dihydroethidium (DHE) staining of 4T1 cells after the indicated treatments. RT dose: 4 Gy. TPEPy-I: 100 μg/mL.


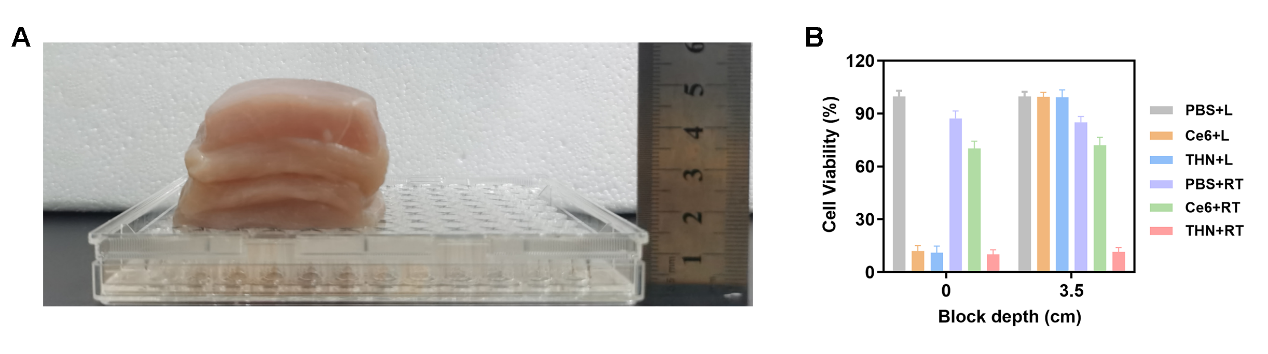


**Figure S12.** (A) Illustration of chicken breast blocking during CCK-8 assay. (B) Cell viability of 4T1 cells after different treatments with the absence or presence of chicken breast blocking. TPEPy-I and Ce6 concentration: 100 μg/mL. RT dose: 4 Gy, L: white light laser, 400-700 nm, 0.1 W/cm^2^, 10 min.


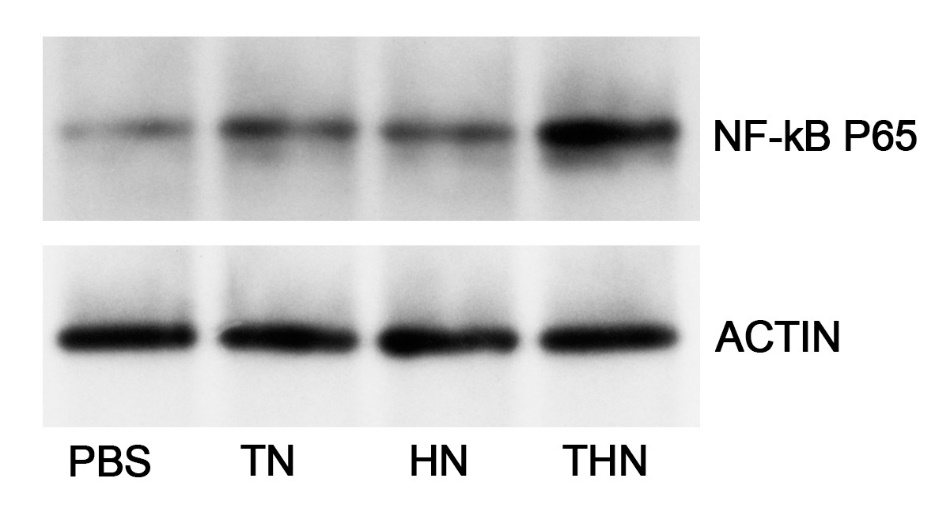


**Figure S13.** Western blot assay of NF-*𝜅*B p65 expression in the RAW264.7 macrophages incubation with indicated treatments. TPEPy-I: 100 μg/mL.


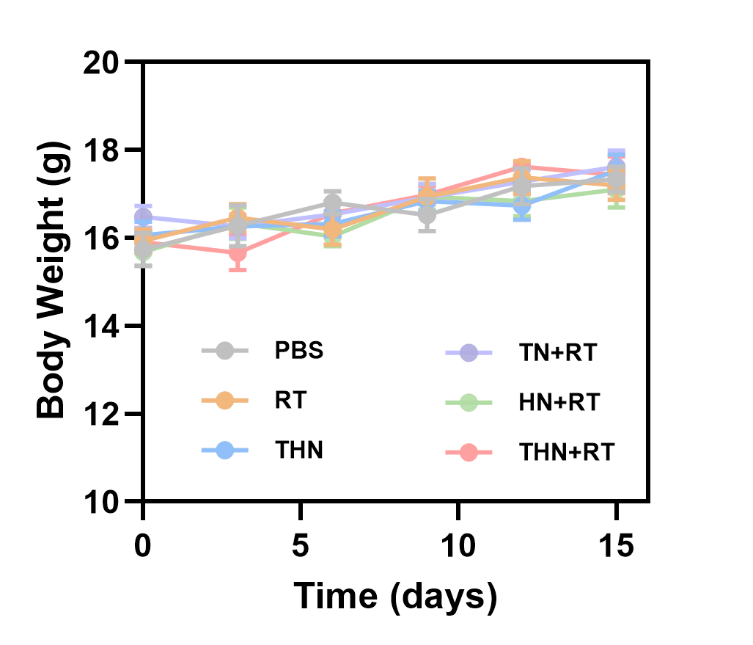


**Figure S14.** Body weight measured every 3 days across all groups.


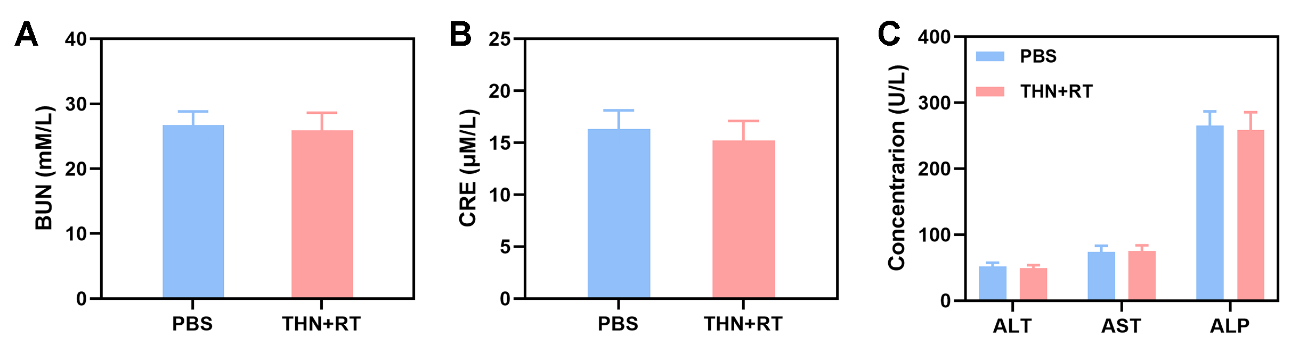


**Figure S15.** The kidney function markers: BUN and CRE, and the liver function markers: ALT, ALP, and AST from mice after different treatments. Data are displayed as the mean±SD (n = 5).


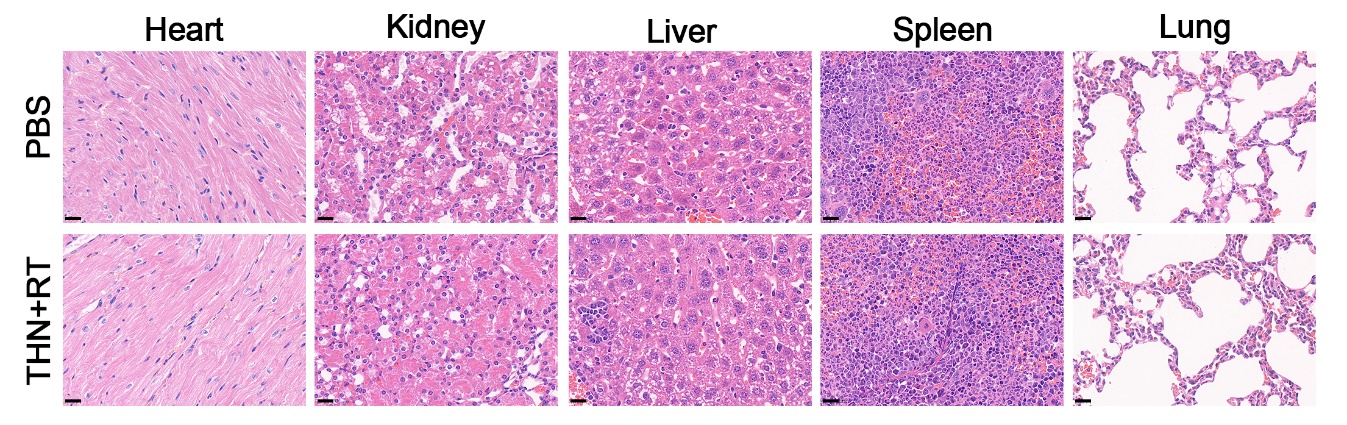


**Figure S16.** HE-stained images for the major organs of mice, including the heart, lung, liver, kidneys, and spleen from mice after different treatments. Scale bars: 20 µm.


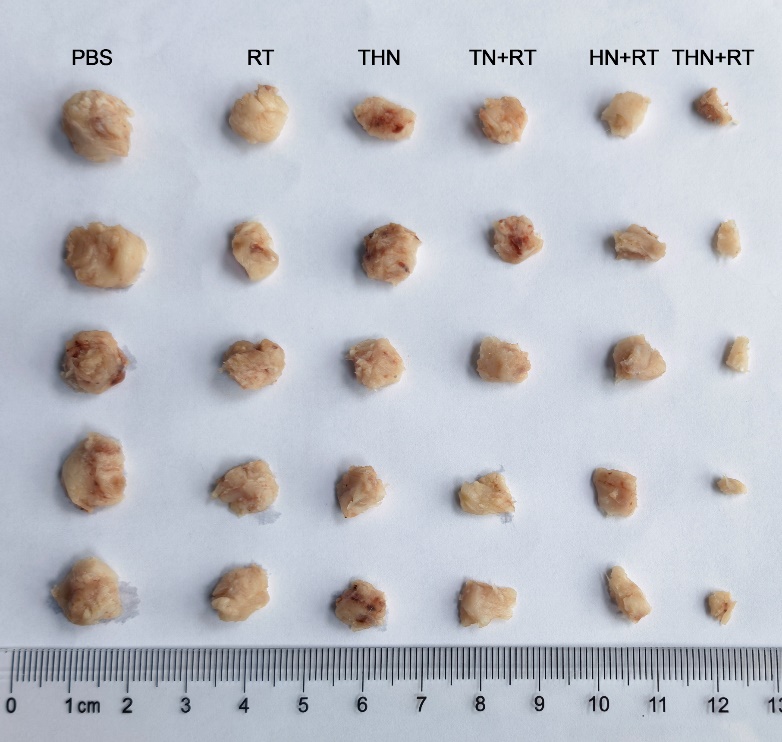


**Figure S17.** Tumor photograph after different treatment.

**References**

[1] W. Xiong, L. Wang, X. Chen, H. Tang, D. Cao, G. Zhang, W. Chen, *Journal of materials chemistry. B* **2020**, 8, 5234.

[2] J. Allison, K. Amako, J. Apostolakis, H. Araujo, P. A. Dubois, M. Asai, G. Barrand, R. Capra, S. Chauvie, R. Chytracek, G. A. P. Cirrone, G. Cooperman, G. Cosmo, G. Cuttone, G. G. Daquino, M. Donszelmann, M. Dressel, G. Folger, F. Foppiano, J. Generowicz, V. Grichine, S. Guatelli, P. Gumplinger, A. Heikkinen, I. Hrivnacova, A. Howard, S. Incerti, V. Ivanchenko, T. Johnson, F. Jones, T. Koi, R. Kokoulin, M. Kossov, H. Kurashige, V. Lara, S. Larsson, F. Lei, O. Link, F. Longo, M. Maire, A. Mantero, B. Mascialino, I. McLaren, P. M. Lorenzo, K. Minamimoto, K. Murakami, P. Nieminen, L. Pandola, S. Parlati, L. Peralta, J. Perl, A. Pfeiffer, M. G. Pia, A. Ribon, P. Rodrigues, G. Russo, S. Sadilov, G. Santin, T. Sasaki, D. Smith, N. Starkov, S. Tanaka, E. Tcherniaev, B. Tomé, A. Trindade, P. Truscott, L. Urban, M. Verderi, A. Walkden, J. P. Wellisch, D. C. Williams, D. Wright, H. Yoshida, *IEEE Transactions on Nuclear Science* **2006**, 53, 270.

[3] J. Sempau, J. M. Fernández-Varea, E. Acosta, F. Salvat, *Nuclear Instruments and Methods in Physics Research Section B: Beam Interactions with Materials and Atoms* **2003**, 207, 107.
